# Land conversion to cropland homogenizes variation in soil biota, gene assemblages, and ecological strategies on local and regional scales

**DOI:** 10.1093/ismejo/wraf264

**Published:** 2025-12-01

**Authors:** Haidong Gu, Zhuxiu Liu, Song Liu, Xiaojing Hu, Zhenhua Yu, Yansheng Li, Lujun Li, Yueyu Sui, Jian Jin, Xiaobing Liu, Zhongjun Jia, Lei Sun, Jonathan M Adams, Marcel G A van der Heijden, Junjie Liu, Guanghua Wang

**Affiliations:** State Key Laboratory of Black Soils Conservation and Utilization, Key Laboratory of Mollisols Agroecology, Northeast Institute of Geography and Agroecology, Chinese Academy of Sciences, Harbin 150081, PR China; Department of Plant and Microbial Biology, University of Zurich, Zurich 8008, Switzerland; State Key Laboratory of Black Soils Conservation and Utilization, Key Laboratory of Mollisols Agroecology, Northeast Institute of Geography and Agroecology, Chinese Academy of Sciences, Harbin 150081, PR China; Department of Plant and Microbial Biology, University of Zurich, Zurich 8008, Switzerland; State Key Laboratory of Black Soils Conservation and Utilization, Key Laboratory of Mollisols Agroecology, Northeast Institute of Geography and Agroecology, Chinese Academy of Sciences, Harbin 150081, PR China; State Key Laboratory of Black Soils Conservation and Utilization, Key Laboratory of Mollisols Agroecology, Northeast Institute of Geography and Agroecology, Chinese Academy of Sciences, Harbin 150081, PR China; State Key Laboratory of Black Soils Conservation and Utilization, Key Laboratory of Mollisols Agroecology, Northeast Institute of Geography and Agroecology, Chinese Academy of Sciences, Harbin 150081, PR China; State Key Laboratory of Black Soils Conservation and Utilization, Key Laboratory of Mollisols Agroecology, Northeast Institute of Geography and Agroecology, Chinese Academy of Sciences, Harbin 150081, PR China; State Key Laboratory of Black Soils Conservation and Utilization, Key Laboratory of Mollisols Agroecology, Northeast Institute of Geography and Agroecology, Chinese Academy of Sciences, Harbin 150081, PR China; State Key Laboratory of Black Soils Conservation and Utilization, Key Laboratory of Mollisols Agroecology, Northeast Institute of Geography and Agroecology, Chinese Academy of Sciences, Harbin 150081, PR China; State Key Laboratory of Black Soils Conservation and Utilization, Key Laboratory of Mollisols Agroecology, Northeast Institute of Geography and Agroecology, Chinese Academy of Sciences, Harbin 150081, PR China; State Key Laboratory of Black Soils Conservation and Utilization, Key Laboratory of Mollisols Agroecology, Northeast Institute of Geography and Agroecology, Chinese Academy of Sciences, Harbin 150081, PR China; State Key Laboratory of Black Soils Conservation and Utilization, Key Laboratory of Mollisols Agroecology, Northeast Institute of Geography and Agroecology, Chinese Academy of Sciences, Harbin 150081, PR China; Heilongjiang Academy of Black Soil Conservation and Utilization, Heilongjiang Academy of Agricultural Sciences, Harbin 150086, PR China; School of Geography and Ocean Science, Nanjing University, Nanjing 210023, PR China; Department of Plant and Microbial Biology, University of Zurich, Zurich 8008, Switzerland; State Key Laboratory of Black Soils Conservation and Utilization, Key Laboratory of Mollisols Agroecology, Northeast Institute of Geography and Agroecology, Chinese Academy of Sciences, Harbin 150081, PR China; State Key Laboratory of Black Soils Conservation and Utilization, Key Laboratory of Mollisols Agroecology, Northeast Institute of Geography and Agroecology, Chinese Academy of Sciences, Harbin 150081, PR China

**Keywords:** soil biota, KEGG gene function, ecological strategy, homogeneity, mollisols

## Abstract

It is widely considered that conversion of natural landscapes to agriculture results in biotic homogenization. A recent study comparing soil biota of 27 paired natural steppe soil (NS) and agricultural soil (AS) sites across 900 km in north-eastern China found that conversion to agriculture had increased spatial gradients in soil functional genes. Using the same shotgun metagenome samples, and bacterial amplicon data, we instead analyzed total observed variation at the between-site and within-site level. We found that from the perspective of community taxonomic composition, archaeal and fungal community variation was decreased in AS compared to NS at both within- and between-site scales. In contrast, the bacterial and metazoal community was homogenized only at the local scale. Total functional KEGG gene assemblage was homogenized in AS at both the local and regional scale, whereas “Y-A-S” strategies in bacteria were homogenized at the local scale but not the between-site scale. Overall, these results show a clear homogenizing effect of agriculture with respect to multiple aspects of soil taxonomic and functional diversity, though varying by scale. Certain abiotic soil properties showed homogenization in AS at within-site and between-site scales may explain this homogenization, and uniformity of plant cover in croplands likely contribute to the effect. These findings confirm and extend global-scale studies showing homogenization of soil biota in agricultural environments, revealing that effects extend to functional genes and the broad taxonomic spectrum of life—with potential loss of soil ecosystem resilience to environmental change resulting from agriculture.

## Introduction

Humans exert a pervasive and increasing influence on the world’s ecosystems, frequently converting natural landscapes into agricultural fields or cityscapes [1]. From the perspective of plants and animals, these human-modified environments are typically characterized by a loss of native biodiversity and a reduction in beta diversity across sites. They are increasingly dominated by cultivated crops, domesticated animals, and a limited suite of ruderal plants and generalist species that thrive under the uniform conditions created by intensive human management [2, 3]. Given the fundamental importance of soil services to both natural and anthropogenic ecosystems, there is considerable interest in understanding the degree of homogenization and simplification of soil biota as a result of conversion from natural to anthropogenic landscapes [4].

Agricultural ecosystems are characterized by frequent human intervention that impose various environmental stressors on soil microorganisms [5, 6]. These stressors include the application of chemical fertilizers, pesticides, as well as alterations in tillage practices [7–9]. In response to these challenges, soil microbial communities are expected to undergo shifts in their functional potential genes [7, 10]. Specifically, these functional potential genes should shift towards adapting the system to environmental stress in agricultural ecosystems. This adaptive capacity is crucial for maintaining the resilience and functionality of soil microbial communities, which in turn support the overall health and productivity of agricultural systems [11].

Beyond its ecological significance, the spatial heterogeneity of soil biota carries important implications for environmental change adaptation [12]. Soil communities collectively form a functional reservoir that could safeguard soil processes against future disturbances, such as land use modifications and climate change [13, 14]. However, contemporary agricultural intensification may undermine this adaptive capacity through biotic homogenization, a process that erodes microbial spatial diversity and biogeographic patterns essential for ecosystem flexibility [1, 15]. This loss of microbial spatial diversity threatens to reduce agroecosystem buffering capacity against emerging environmental stresses [16, 17], underscoring the importance of preserving microbial biogeographic patterns for sustainable soil management [18, 19].

Soil bacterial communities contain vast functional genetic diversity, much of it poorly characterized. This hinders progress in predicting soil microbial responses to environmental change [20]. The life history strategy framework has proven effective for comparing broad organismal strategies [21], critical gaps persist in understanding microbial trait associations. Although plant life history strategies are well defined across established trait dimensions, analogous frameworks for soil microorganisms remain underdeveloped [22, 23]. Advances in large-scale metagenomic datasets now enable systematic exploration of microbial functional gene diversity, allowing researchers to link community level traits to environmental drivers [24]. For example, a previous study indicated the importance of microbial yield (Y), resource acquisition (A), and stress tolerance (S) traits, concepts adapted from the plant CSR scheme, in regulating soil carbon cycling [25]. These theoretical advances provide testable hypotheses about key traits governing microbial adaptation. Analysis of community aggregated traits (CATs) through metagenomic sequencing offers a powerful approach to detecting shifts in bacterial functional profiles, thereby allowing the application of life history strategy theories to microbial communities [26]. A recent study revealed consistent adjustments in microbial life history strategies under acidic conditions in grassland soils, highlighting their adaptive plasticity. However, critical questions remain unresolved, particularly regarding how these strategies shift during land use change in vulnerable ecosystems such as black soil, where microbial adaptation mechanisms are poorly documented [27].

A recent global study contrasting global cropland soils with nearby areas of natural and semi-natural ecosystems revealed that bacterial communities in agricultural systems are taxonomically homogenized relative to those in natural environments [14]. However, soil biota encompass diverse organismal groups beyond bacteria, and taxonomic composition alone may not fully capture functional aspects of soil communities, such as functional gene profiles, which are critical for understanding biogeochemical processes [28]. Our earlier study compared the spatial trends in turnover of overall KEGG gene functions between natural steppe soils (NSs) and agricultural soils (ASs), in the chernozem zones of north-eastern China, hypothesizing that there would be reduced distance decay amongst the AS soils [13]. Among 27 paired sites, we in fact found that the AS had greater heterogeneity and increased spatial turnover in KEGG gene functions. We proposed that this pattern might be due to disrupted homeostatsis in the agricultural system resulting from loss of the insulating influence of the continuous grassland cover of the steppe.

In the present study, we adopted a complimentary approach to the same dataset, focusing on the overall variation of communities amongst the total set of sites, in terms of taxonomic composition, total functional gene assemblage, and bacterial ecological strategies based on functional genes. Unlike our previous analysis, which emphasized the slope of the spatial distance decay relationships, this study examines the scatter of points that this trend line passes through, encompassing the total variation in soil biota composition observed in the dataset of sites [13]. This approach offers a distinct yet integrative perspective by incorporating both functional genes and taxonomic composition, aspects not fully covered in our earlier study. We hypothesized that: (i) AS would exhibit homogenization of taxa and their functional genes compared to natural soil; (ii) conversion from NS to AS would enhance the stability of soil biotic communities; and (iii) the functional potential genes would shift towards resistance to adapt to the environmental stress in agricultural ecosystems. This work aims to deepen our understanding of how land-use conversion affects soil biota and to inform strategies for sustainable soil management.

## Materials and methods

### Soil samples collection, soil physicochemical property, and soil enzyme activity determination

**Figure 1:**
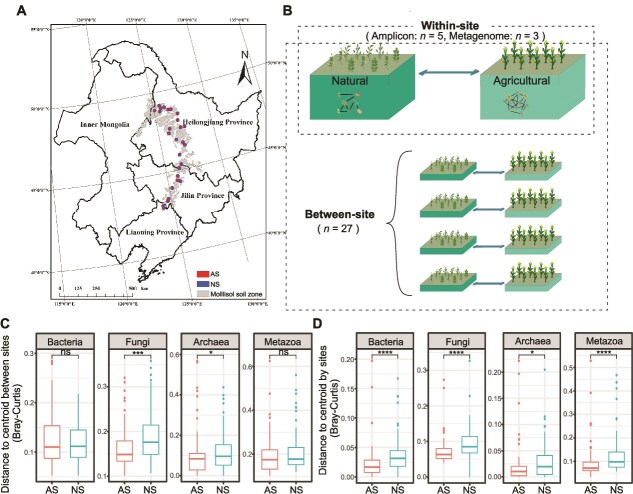
Location of sampling sites and concept of the analysis. (A) The 27 sampling sites are marked in the map. (B) Pairwise-sampling of agricultural (AS) and natural soils (NS). Five soil samples were collected at each land use type at one site. Differences in homogeneity at family level of the soil bacterial, fungal, archaeal, and metazoal communities at the inter-site (C) and the intra-site (D) level between AS and NS. The asterisks indicate the significant difference between those two land use types (^*^P < .05, ^***^P < .001, ^****^P < .0001, NS *P* > .05; Wilcox sum rank test).

Mollisols, a soil type prevalent across extensive areas of temperate semi-arid regions, are predominantly distributed in three provinces of Liaoning, Jilin, and Heilongjiang in northeast China, covering a total area of ~1.09 × 10^6^ km^2^ [29]. Some mollisol areas were already being cultivated 200 years ago, but most areas have been used for agricultural production for ~50–65 years. We selected the remnants of steppe vegetation that had never been used for farming, and adjacent agricultural fields which were used for a maize-corm rotation (http://northeast.geodata.cn/). Soil sampling site locations and chemical fertilization for the AS were summarized in Table S1. A total of 270 topsoil samples (0–15 cm depth) were collected in October 2020 after crop harvest from 27 sites (comprising 5 replicates × 2 soil types), to examine the effects of cropland conversion on bacterial community assembly patterns and life-history strategies (Fig. 1, Table S2). The fundamental details regarding the selection of soil sites and the methodology employed for soil sampling have been previously elucidated in our previous studies [3, 13].

Soil pH, soil total carbon, nitrogen, phosphorus, and potassium (TC, TN, TP, and TK), soil available nitrogen (NH_4_^+^-N and NO_3_^−^-N), soil salt content (EC), and soil available phosphorus and potassium (AP and AK) were determined according to the methodology described in our previous study [13]. Climatic information was extracted from the WorldClim database (www.worldclim.org/data/index.html).

The activities of three principal hydrolytic enzymes including C-degrading enzyme (β-1,4-glucosidase [BG]), P-degrading enzyme (acid phosphatase [ACP]), and N-degrading enzymes (L-leucine aminopeptidase [LAP]), were assessed through standard fluorometric methods with the highly fluorescent compounds of 4-methylumbelliferone and 7-amino-4-methyl-coumarin (Uplc-MS Testing Technology Co., Ltd, Shanghai, China). We meticulously maintained a constant temperature of 25°C throughout the determination process. The concentration of the fluorescent substrate was set at 200 μM, and the reaction time was established at 3 h to ensure optimal assay conditions [30]. Fluorescence intensity was recorded at excitation and emission wavelengths of 365 nm and 450 nm, respectively, using a microplate reader (BMG LABTECH, Germany). The EEAs were subsequently calculated and expressed in units of μmol d^−1^ g^−1^ soil.

### DNA extraction, qPCR, amplicon sequencing, and metagenome sequencing

Soil total DNA was extracted from 0.5 g fresh soil using the E.Z.N.A Soil DNA Kit (OMEGA, USA) according to the procedures of the manufacturer. The quality of the extracted DNA was examined by a NanoDrop 2000 spectrophotometer (Thermo Scientific, USA).

The qPCR assays targeting bacterial [31] and archaeal [32], as well as fungal genes [33] in AS and NS soils, respectively, were carried out in triplicate with LightCycler 480 (Roche Applied Science). The amplification reactions were performed according to the following steps: 6 μl of AceQ SYBR Green Master Mix (Vazyme, Nanjing, China), 0.2 μM of forward and reverse primers, 1 μl of DNA, and nuclease-free water were added to adjust the final volume to 16 μl. The PCR conditions were 95°C for 5 min, followed by 40 cycles of 15 s at 95°C, 30 s at 60°C. A standard curve was established by constructing standard quality particles of different concentrations and determining the copy numbers, which was used to quantify the DNA of 270 soil samples.

The primer pair 515F/907R with unique barcode were used to amplify the V4-V5 region of the soil bacterial 16S rRNA gene. The PCR system and amplification conditions were described previously [34]. The PCR products were further pooled and purified by an agarose gel DNA purification kit (TaKaRa, Dalian, China). The purified PCR products were sequenced on an MiSeq System (Illumina) at Majorbio Bio-Pharm Technology Co., Ltd (Shanghai, China).

The identical DNA extractions employed for amplicon sequencing were concurrently utilized for shotgun metagenomic sequencing. A standard metagenomic library construction method was employed directly on extracted DNA from metagenomes. Paired-end sequencing (2 × 150 bp) was performed using the Illumina Inc. (San Diego, CA, USA) platform. The adapters, which contain the entire sequencing primer of the hybridization sites, were attached to the blunt-end fragments. Metagenomic library was performed on the HiSeq System (Illumina) at Majorbio-Pharm Technology Co., Ltd (Shanghai, China).

### Bioinformatic analysis

The raw 16S rRNA gene sequences were processed to generate amplicon sequence variants (ASV) using Quantitative Insight into Microbial Ecology 2 (QIIME2) [35]. The barcodes, primers, and low-quality sequences (read length < 50 bp or average quality scores < 20) were subsequently removed. The forward and reverse sequences were merged and assigned to each sample based on barcode. The filtered sequences were denoised using DADA2 algorithm [36]. Taxonomy assignments of each ASV were conducted using Naive Bayesian Classifier [37] against SILVA database (Release 138) (https://www.arb-silva.de/). A total of 56 491 ASVs were obtained, with each sample being rarefied to the minimum number (14 601 sequences) required for downstream analyses.

To predict functional genes of bacterial communities, the adapter sequences of raw shotgun metagenomic reads were firstly removed using SeqPrep (https://github.com/jstjohn/SeqPrep). Low-quality reads (read length < 50 bp or average quality scores < 20) were then trimmed with Sickle v1.33 (https://github.com/najoshi/sickle). The clean reads were assembled into contigs using Megahit v1.1.2 [38], and the open reading frames (ORFs) of contigs were then predicted using MetaGene [39]. The predicted ORFs with no less than 100 aa were retained and clustered using CD-HIT v4.7 (http://www.bioinforamatics.org/cd-hit/) with 95% identity and 90% coverage. The number of reads mapping to genes for each sample was calculated using SOAPaligner 2.21 [40]. Taxonomy and KEGG annotations were performed by diamond v0.8.35 [41] against the NR database (ftp://ftp.ncbi.nlm.nih.gov/blast/db) and KEGG (http://www.genomme.jp/kegg/) database with best-hit, e-value 1e^−5^ at “blastp” format. Taxonomy annotated to the bacterial taxa was filtered according to the annotation results of the NR database.

The microbial life history strategies (based on Y-A-S theory) were predicted with Hidden Markov Models (HMMs) using hmmsearch [42]. HMM model was retrieved from the Microtrait Database [43]. Sequence hits with an e-value cutoff score of 1e^−5^ were removed to ensure high confidence in all hits. The profiles of functional genes of bacterial communities were calculated as CPM (counts per million-normalized) according to the methodology described previously [13]. The average genome size (AGS) of soil microbial communities of each sample was estimated using MicrobeCensus v1.1.1 with default parameters (−n 2 000 000, −l 100, −1 -5) [24, 44].

### Statistical analysis

Alpha diversity was assessed by calculating the richness and Shannon-Wiener index for both ASVs and KO genes, using estimate_richness function in phyloseq package [45]. The dispersion of taxonomic and functional gene communities for each group in AS and NS, as well as the dispersion of soil chemical properties, were calculated based on Bray–Curtis distance matrix with betadisper R function to evaluate the homogenization effect in regional scales [46]. This method also calculated in local scales which was grouped by site in AS and NS. The distance-decay relationships (DDRs) were calculated to evaluate the distribution patterns of bacterial ASVs and functional genes between the geographic distances (Euclidean distances) and community similarities (1-dissimilarity of the Bray–Curtis distance metric). A linear regression was employed to relate the geographic distances and the Bray–Curtis distances.

A variation-partitioning analysis (VPA) was conducted to disentangle the relative importance of environmental factors and spatial factors on the variation in bacterial and functional gene communities [47]. Spatial variables were derived from geographic distances using Moran’s eigenvector maps, also known as the principal coordinates of neighbor matrices (PCNM) algorithm, which was able to deconvolute total spatial variation into a discrete set of explanatory spatial scales [48]. Forward selection procedures were subsequently employed to select respective subsets of environmental and spatial variables. The forward selection procedure was terminated if the significance level (*P* > .05) was reached or if no improvement in the selection criterion (${R}^2$) was observed upon the additional any variables. Subsequently, a two-way permutational multivariate analysis of variance (PERMANOVA) was performed using the selected variables. The effect of species sorting is represented by pure environmental variation without a spatial component, whereas the effect of dispersal limitation is represented by pure spatial variation without an environmental component. The fractions of explained variance are based on adjusted fractions (${R}_{adj}^2$, adjusted coefficient of multiple determination), which accounts for the number of variables and sample sizes.

The Sloan neutral community model (NCM) was employed to determine the potential importance of stochastic processes to the community assembly [49]. In the model, the estimated migration rate is a parameter for evaluating the probability that a random loss of an individual in a local community would be replaced by dispersal from the metacommunity. A higher m value indicates that microbial communities are less dispersal limited [50]. To reveal the patterns of deterministic ecological processes, we estimated Levins’ niche breath (B) index [51, 52] using the spaa R package. In addition, the normalized stochasticity ratio (NST) was quantified using the R package NST, to identify the relative contribution of deterministic and stochastic processes in driving soil bacterial and their functional gene assembly. NST is an index developed with 50% as the boundary point between more deterministic (<50%) and more stochastic (>50%) assembly [53].

To reveal the intricate interaction patterns, a co-occurrence network was constructed based on Pearson correlation with netET R package (https://github.com/Hy4m/netET). The analysis included bacterial ASVs with a relative abundance exceeded 0.01% that were present in at least 25% of samples within the specific habitat. The node and network properties were calculated by igraph R package [54]. The topological role of each node was determined by calculating within-module connectivity (*Zi*) and among-module connectivity (*Pi*). Nodes were categorized as follows: module hubs were defined as those with Zi > 2.5 and *Pi* < 0.62; connectors as those with *Zi* < 2.5 and *Pi* > 0.62; and peripherals as those with *Zi* < 2.5 and *Pi* < 0.62 [55]. Additionally, nodes were ranked according to the standardized z-scores of node degree and betweenness centrality, and the top 5% were classified as network hubs [56]. Nodes identified as either module hubs or network hubs were collectively designated as hub nodes. The networks were visualized using ggraph R package (https://github.com/thomasp85/ggraph).

## Results

### Changes in soil microbial abundance and enzyme activity under different land use types

Microbial abundances in 27 paired NS and AS were determined via real-time PCR, and C-, N-, and P-degrading enzyme activities (BGC, LAP, and ACP) were measured using standard fluorometric methods. Bacterial abundance in AS ranged from 2.01 × 10^10^ to 1.10 × 10^11^ copy g^−1^ dry soil, and from 9.77 × 10^9^ to 1.01 × 10^11^ copy g^−1^ dry soil in NS. Fungal abundance was 5.05 × 10^6^ to 1.66 × 10^8^ copy g^−1^ in AS, and 1.32 × 10^7^ to 3.16 × 10^8^ copy g^−1^ in NS. Archaeal abundance was 2.09 × 10^5^ to 1.18 × 10^7^ copy g^−1^ dry soil in AS, and 4.83 × 10^5^ to 9.24 × 10^6^ copy g^−1^ in NS. The bacterial and archaeal abundance was significantly higher in AS than that in NS, while the fungal abundance showed a different pattern (Figs S1A–C).

Activities of the C-, N-, and P-degrading enzymes (BGC, LAP, and ACP) were significantly lower in AS than in NS (Figs S1D–F). Correspondingly, strong correlations (*P* < .05) were observed between the abundances of genes involved in carbon-, nitrogen-, and phosphorus-degradation pathways and the activities of their respective enzymes in both AS and NS (Fig. S2).

### Bacterial and functional gene communities and environmental drivers

Bacterial communities differed significantly between AS and NS. Approximately 50% of core ASVs showed significant abundance shifts between these ecosystems. Specifically, 473 and 401 core ASVs were identified in AS and NS, respectively, with 353 ASVs being shared (Fig. S3A). Most core ASVs were affiliated with the phyla Pseudomonadota, Acidobacteriota, Actinomycetota, and Bacteroidota, and their relative abundances varied distinctly with latitude (Fig. S3B). A subset of 25 bacterial genera (including taxa from Acidobacteriota, Actinomycetota, Myxococcota, and Pseudomonadota) exhibited higher abundance in AS adjacent to NS, suggesting a taxon-specific and adaptive response to agricultural practices (Fig. S3C).

**Figure 2:**
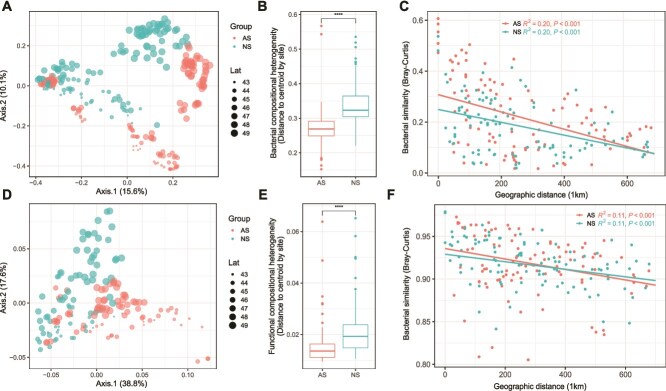
The community structures in the soil bacterial taxa and their functional genes. Principal Coordinates Analysis (PCoA) shows the beta-diversity using Bray–Curtis distance (A, D). Adonis test: *P* < .001, *R* = 0.5. Points were scaled by latitude. The soil community composition heterogeneity of soil bacteria (B) and KO genes (E) in AS and NS. Distance-decay relationships (DDRs) between the similarity of the bacterial and function gene communities and geographic distance (C, F). Red and blue color represents AS and NS, respectively. Boxes include median and 25th/75th percentile of the distances to the group centroid derived from betadisper (vegan R package). Asterisks indicate significant differences in compositional heterogeneity based on Wilcox sum rank test (^***^P < .001).

RDA showed soil pH and TC as the key drivers of bacterial ASVs in both AS and NS (Fig. S4A), a result corroborated by RF analysis (Fig. S4B). Correlation analysis revealed specific ASVs associated with these parameters (Table S3). In AS, 368 ASVs were positively and 179 were negatively correlated with TC, and 169 favored high-pH and 230 favored low-pH conditions. In NS, 163 ASVs were positively and 322 were negatively correlated with TC, and 163 and 196 ASVs favored high- and low-pH conditions, respectively (Fig. S4C). Building on a previous study of land use effects on core genes [13], we analyzed the drivers of the overall functional gene profile (Table S4). RDA demonstrated soil pH and TC were also the key drivers shaping the overall functional gene assembly in AS and NS, respectively (Fig. S5A), which was confirmed by RF analysis (Figs. S5B). Correlation analysis showed that, in AS, 284 genes were positively and 307 were negatively correlated with TC, and 544 and 732 genes preferred high- and low-pH conditions. In NS, 356 genes correlated positively and 584 negatively with TC, and 327 and 423 genes were associated with high- and low-pH conditions, respectively (Figs. S5C).

### Impact of land use on the taxonomic and functional gene assemblages

Metagenomic analysis revealed a higher degree of homogenization in soil biota in AS across multiple dimensions, compared to NS (Fig. 1C and D). Specially, bacterial and metazoal communities exhibited homogenization primarily at local scales (for both bacteria and metazoa: *P* < .0001). However, no significant effects were observed at regional scales in AS (bacteria: *P* = .91; metazoa: *P* = .087). In contrast, fungal and archaeal communities showed homogenization at both local (fungi: *P* < .0001; archaea: *P* = .019) and regional scales (fungi: *P* < .001; archaea: *P* = .040) (Fig. 1C and D). Furthermore, total KEGG functional genes (Table S5) across the major kingdoms of life revealed that bacteria, fungi, archaea, and metazoa exhibited homogenization patterns at both local (Figs S6E–H; bacteria: *P* < .0001; fungi: *P* < .0001; archaea: *P* < .0001; metazoa: *P* = .014) and regional scales (Figs S6A–D; bacteria: *P* = .042; fungi: *P* = .017; archaea: *P* = .020; metazoa: *P* = .043) in AS. Consistent with these findings, amplicon sequencing of bacterial 16S rRNA gene also demonstrated community homogenization in AS, evident both within and between sites (Fig. S7; within sites: *P* < .0001; between sites: *P* < .001).

Soil chemistry in AS was also found to be homogenized at a local scale (Fig. S8B), with no significant homogenization observed at a regional level (Fig. S8A). Specifically, total nitrogen, ammonia nitrogen, electrical conductivity (EC), total potassium, and available potassium exhibited significantly greater homogeneity between AS sites (Fig. S9). Conversely, nitrate nitrogen and available phosphorus showed contradictory trends. Within individual AS sites, total carbon, total nitrogen, total potassium, and available potassium were found to be significantly more homogenous, with the exception of nitrate nitrogen (Fig. S10).

### Structuring, assembly processes and ecosystem networks

Principal coordinate analysis (PCoA) based on Bray–Curtis dissimilarity revealed a clear distinction of bacterial communities (PERMANOVA; *R*^2^ = 0.06, *P* < .001), and soil functional gene communities (PERMANOVA; *R*^2^ = 0.13, *P* < .001) between AS and NS (Fig. 2A and D). The community structure of bacterial taxa and functional genes exhibited greater similarity across the 27 sites distributed in AS than in NS, regardless of spatial variations (*P* < .001, Fig. 2B and E). The distinct pattern of DDRs was evident in both AS and NS (*P* < .001, Fig. 2C and F), and the steeper DDR slopes for the similarity of both bacterial taxa and functional genes in AS than in NS indicated a stronger influence of spatial proximity on community composition in AS.

**Figure 3:**
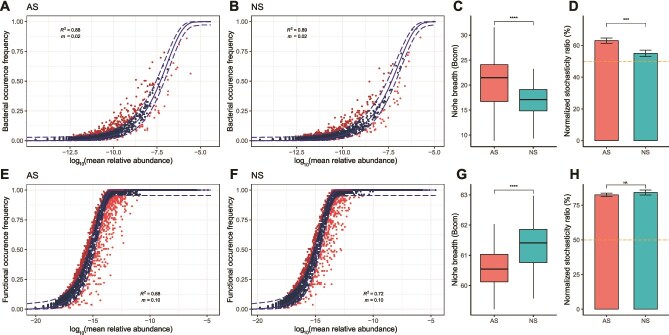
Assembly mechanisms of soil bacterial communities and their functional gene communities in AS and NS. Fit of the neutral community model (NCM) of soil bacterial communities and their functional gene communities in AS (A, B) and NS (E, F). Niche breadth (*B*com) analysis of soil bacterial communities and their functional gene communities (C, G). Normalized stochasticity ratio (NST) analysis of soil bacterial communities and their functional gene communities (D, H). Asterisks indicate significant differences between AS and NS based on Wilcox sum rank test (^***^P < .001, ^****^P < .0001).

A series of theoretical models were constructed to assess the impact of assembly processes on bacterial and functional ecological communities (Fig. 3). The *R*^2^ values of the NCMs were 0.88 and 0.89 for bacterial communities (Fig. 3A and B), and 0.68 and 0.72 for functional gene communities in AS and NS, respectively (Fig. 3E and F), showing that the assemblage of both bacterial and functional gene communities in each habitat was well explained by the neutral theory.

Community-level habitat niche breadths (*B*com) revealed that bacterial communities exhibited a 19.2% greater niche breadth in AS compared to NS, while the functional gene community in AS had a 1.2% lower niche breadth than in NS (Fig. 3C and G). The normalized stochasticity ratio based on Bray–Curtis distance (NST_bray_) index showed that the bacterial and functional gene communities in both AS and NS were predominantly governed by stochastic processes (NST_bray_ > 50%). In particular, the bacterial communities in AS (NST_bray_ = 0.63) appeared to be more stochastic than in NS (NST_bray_ = 0.55), while the functional gene communities in AS (NST_bray_ = 0.82) were similar to those in NS (NST_bray_ = 0.84, Fig. 3D and H).

**Figure 4:**
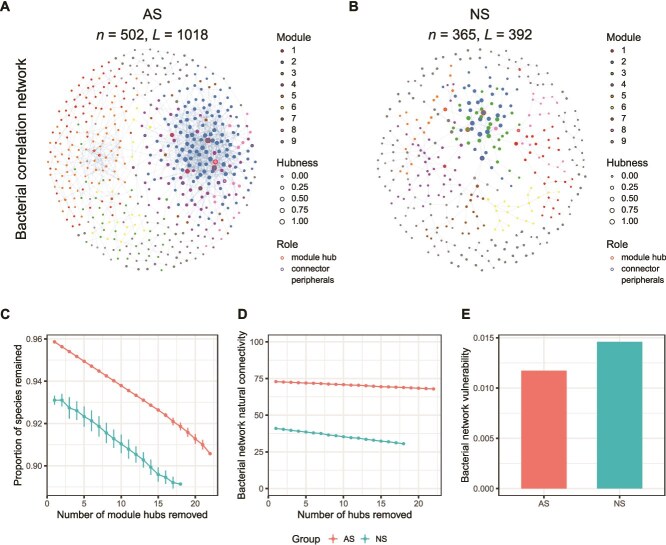
Microbial environmental networks and stability metrics based on network features. Co-occurrence networks for revealing the potential linkages among bacterial communities in AS (A) and NS (B). A connection stands for a strong (correlation coefficients > 0.7) and significant correlation (*P* < .01). The color indicates different network modules. The size of node is the hubness index. The color of circles around the nodes indicates the role of nodes in the network according to *Zi*, *Pi* value. Differences of stability metrics between AS and NS (C, D, and E).

Co-occurrence networks were constructed to elucidate the patterns of correlations of bacterial taxa in AS and NS. The networks showed that AS had a greater number of nodes and linkages compared to NS for bacterial taxa (Fig. 4A and B, Table S6). The variation in stability and the resistance of bacterial networks by removing the hubs indicated that agricultural intensification enhanced the robustness of the ecosystem network but reduced its vulnerability (Figs 4C–E). The topological properties of the network structures in bacterial communities showed a higher number of key nodes in AS (Table S7) compared to NS (Table S8), indicating a greater ecosystem stability in soil bacterial communities in AS than in NS.

### AS showed greater local homogeneity of Y-A-S gene community structures

**Figure 5:**
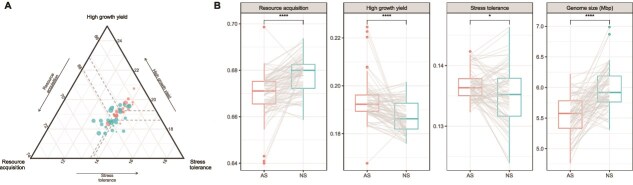
The comparison of bacterial life history strategies of soil functional gene communities in AS and NS. Ternary plots representing the composition of the bacterial life history strategies (A). Boxplots indicate significant differences on relative abundances of the bacterial life history genes and average genome sizes between AS and NS (B). Asterisks indicate significant differences in compositional heterogeneity based on Wilcox sum rank test (^*^P < .05, ^****^P < .0001).

In order to gain a deeper understanding of the impact of land use change on the ecological adaptations of soil bacteria, we employed the Y-A-S theoretical framework to estimate the history strategies of soil bacterial communities (Fig. 5). The relative abundance of functional genes associated with high growth yield (Y) and stress tolerance (S) was significantly increased by agricultural practice, whereas the relative abundance of genes associated with resource acquisition (A) was decreased. Furthermore, it was observed that the AGS of soil bacteria decreased in AS (Fig. 5B).

A and S strategy exhibited significant homogenization in AS at the local scale (Figs S11D–F; A: *P* < .0001; Y: *P* = .080; S: *P* = .0004). There was no significant homogenization at the regional scale (Figs S11A–C; A: *P* = .183; Y: *P* = .055; S: *P* = .336). The AGS of soil bacteria increased as soil pH shifted from acidic to neutral in AS (Fig. S12A). This was also significantly associated with MAP (Fig. S12B) and MAT (Fig. S12C). AGS in AS was influenced by a broader spectrum of soil chemical properties than in NS. Specifically, it correlated positively with total carbon, total nitrogen, the C/N ratio, and total phosphorus, but negatively with nitrate nitrogen and available phosphorus. In contrast, AGS in NS remained largely independent of these chemical properties as well as MAT and MAP (Fig. S13). Furthermore, AGS of soil bacteria was found to be positively correlated with resources acquisition (Fig. S14A; AS: *R*^2^ = 0.21, *P* < .001; NS: *R*^2^ = 0.08, *P* = .009), but negatively correlated with growth yield in AS (Fig. S14B; AS: *R*^2^ = 0. 28, *P* < .001; NS: *R*^2^ = 0.06, *P* = .027).

## Discussion

### Shifts in homogeneity of soil biota resulting from land use conversion

This study demonstrated a clear trend towards homogenization under agriculture (AS) in both community taxonomic composition and in functional gene composition, for major categories of soil life (Fig. 1). For bacteria, archaea, fungi, and metazoans, family level taxonomic community composition was homogenized at the inter-site and the intra-site level (Fig. 1C and D), whereas for bacterial amplicon data showed homogenization under AS down to the level of ASVs (Fig. S7). Through reducing taxonomic turnover, AS also results in overall reduced taxonomic beta diversity of the major kingdoms of life at the taxonomic family level and at the ASV level for bacteria. This is against a background in which taxonomic alpha diversity of each group is lower under AS compared to NS.

This general pattern also held true in terms of KEGG functional gene homogeneity at intra-site and inter-site scales, for each of these groups (Fig. S6). Based on ecological interpretation of KEGG gene assemblages, the Y-A-S strategy position of bacterial communities is likewise homogenized at the intra-site level but not the inter-site level (Fig. S11). These findings align on a regional scale with a global-scale study by the previous study, where soil bacterial communities were compared at 44 paired natural vegetation and agricultural sites [14]. In this study, however, the comparison extends to multiple kingdoms of life in terms of the taxonomic perspective, an adds the functional gene perspective from using KEGG functions in the metagenome, together with Y-A-S ecological strategies in the case of bacterial KEGG functions. As such, it emphasizes how pervasive the effects of land use conversion to agriculture on soil biota homogeneity actually are. This agrees with the major trend towards taxonomic homogenization seen globally in bacterial communities with conversion from natural habitat to agriculture [57], as well as that seen for other groups such as plants [58], birds [59], and small mammals [60].

### Spatial homogeneity in community composition and function is greater in AS than in NS

The observed of homogenization of variation in biota, gene assemblages, and ecological strategies in AS contrasts with findings from our earlier study [13], which found that the slope of distance decay of soil biota similarity was in fact increased in AS relative to NS, contrary to our original hypothesis that the slope of AS would be shallower. This increased distance decay was suggested as possibly due to reduced homeostasis in the soil community under AS in relation to background variation in climate [2]. However, on further reflection our earlier study was incomplete in the sense that it concentrated only on the regression line of distance decay in soil biota similarity, and ignored the total scatter of soil communities amongst the individual sites along that line. Furthermore, our earlier study did not consider variation between replicate cores within each site, which would offer an additional local-scale perspective. As such, by encompassing the full range of variation in soil biota amongst sites, rather than only the distance trend, the present study offers a complimentary and possibly more meaningful perspective on the effects of land use conversion on heterogeneity in soil biota. By systematically quantifying β-diversity across taxonomic, functional, and strategic dimensions, the present study resolves this apparent contradiction, demonstrating that agricultural conversion simultaneously increases spatial turnover rates (steeper distance-decay slopes), while reducing total compositional heterogeneity (lower overall β-diversity), a duality explained by the amplification of localized environmental filtering under cultivation.

We suggest that the conversion of steppe to cropland may induce a change in trait-based filtering, leading to the displacement of certain bacterial taxa and a consequent reduction in overall diversity, and favoring those microorganisms that are more adept at thriving in agricultural environments [61]. The greater stochasticity observed in AS results from frequent temporal disturbances. These arise partly from routine farming practices, such as plowing, planting, pesticide application and fertilization, as well as from amplified fluctuations in temperature and moisture [8]. The latter occurs because the soil is directly exposed to weather and sunlight, lacking the continuous insulating cover of steppe vegetation and plant litter. This will lead to unpredictable recolonization and priority effects in the soil community, as the most favored niches constantly change. This will lead to unpredictable recolonization and priority effects in the soil community as the most favoured niches constantly change—the system is in a constant state of disruption and adjustment, which leads to random priority effects of species populations newly favoured by a shift in conditions [62]. Thus, although the agricultural landscape is spatially homogenized, recurring fine-scale temporal disturbances amplify stochasticity at the community level [63]. Overall, this spatially consistent but fluctuating selective pressure may result in a more homogenized community i.e. adapted to withstand the perturbations of human activities [64]. The homogeneity of soil bacterial communities in AS, coupled with a wider niche breadth (Fig. 3), may enhance the robustness of bacterial communities in the face of anthropogenic disturbances (Fig. 4). The broader niche breadth of bacteria, indicative of greater metabolic plasticity, is underpinned by a diverse array of functional genes. This suggests that, despite being functionally more constrained by the environment, AS harbor a rich tapestry of capabilities that enable them to adapt to the challenges posed by human intervention [65].

### Possible mechanisms behind homogenization in the agricultural system

It is plausible that conversion to agriculture homogenizes soil biota by spatially homogenizing various soil characteristics [66]. Conversion of natural land to agriculture tends to involve levelling the land surface (destroying within-site microtopography and between-site differences in slope angle and aspect), adding drainage when water content is high while irrigating when water is deficient, adding chemical fertilizers and pesticides in large and fairly uniform quantities, and adding lime where necessary to help achieve a certain desirable pH for crop productivity [67, 68]. Comparing the range of site-to-site and within-site variability in soil characteristics with this dataset shows that indeed there is greater uniformity in at least some soil factors at the site-to-site and within-site level (Fig. S9 and S10). Under agriculture, this may select for a more consistent assemblage of species whose niche requirements overlap with the more consistent environmental conditions, or likewise for individual genes whose functions are confined to a particular environmental range (Fig. 3).

Adding to this homogeneity in abiotic environment is likely the homogeneity in terms of plant cover. Instead of the diverse and heterogenous plant cover of steppe, agricultural fields have a far more limited and more consistent range of plant species. This will give a narrower range of different potential interactions which may support or exclude microbial species, genes, and strategies [69]. Our study did not directly measure aspects of the soil functionality or resilience under AS compared to NS. Conversion to AS in itself is likely to result in large changes in soil functionality as a result of changes in nutrient regime, cultivation and plant cover, compared to NS [70]. Network analysis of bacterial community shows that by several different measures, the AS is in fact predicted to have greater stability on average than NS (Fig. 4). This may be seen as product of the taxonomic simplification of soil community under a regime of high fertilizer and pesticide inputs, and frequent cultivation, in AS [68].

Biotic homogenization in AS may critically constrain the ecosystem’s reserve capacity to buffer against environmental stressors such as climate change and pollution. A potential mechanism underlying this reduced resilience involves evolutionary trade-offs in microbial genome architecture, as conceptualized by the Black Queen hypothesis [71]. This framework helps explain our observation of decreased average bacterial genome sizes in AS under acidic conditions (Fig. 5, compared to neutral-pH natural soils, NS). Larger genomes, while encoding greater functional diversity, impose higher metabolic costs for gene maintenance and expression with a liability under environmental stress [72]. Agricultural intensification appears to amplify this selection pressure, driving genome streamlining as observed in acid-adapted ecosystems [73, 74]. We further identified climate-mediated selection on genome size, with mean annual temperature (MAT) and precipitation (MAP) showing stronger correlations with bacterial genome metrics in AS than NS (Fig. S12B and C). In contrast, NS exhibits higher ecological stability, with no significant correlations observed between genome size and pH, as well as MAT and MAP, suggesting that buffered microbial communities experience weaker environmental filtering. This finding heightened sensitivity may reflect the breakdown of soil aggregate structures under tillage, and of the insulating “blanket” of steppe vegetation above ground, exposing microorganisms to intensified climatic fluctuations (e.g. thermal extremes, moisture variability). Such exposure aligns with evidence that warmer conditions disproportionately select against large genomes due to elevated metabolic demands [75, 76]. In terms of the Y-A-S theory, a reduction in genome size enables bacteria to enhance their potential functions related to growth and yield to adapt to disturbed environments. However, this comes at the cost of reduced resource acquisition.

## Conclusion

The larger overall spatial variability of taxa, KEGG gene types, and Y-A-S ecological strategies seen in the NS samples may preserve a greater range of potential responses and interactions, which could be recruited under future, changed conditions including future climates or modified cropping systems. Fragments of the original natural habitat, such as those sampled in this study, may thus serve as reservoirs of soil biodiversity, potentially supplying adjacent agricultural lands with taxa and gene functions under changing conditions. The clear homogenization under agriculture contrasts with our previous conclusion based on DDR slopes, which indicated steeper spatial turnover in AS and suggested functional diversification at the gene functions [13]. We regard a focus on the total “cloud” of variation, rather than the linear distance trend, as a better measure of the pool of variability in microbial biota. The current study also incorporated taxonomic composition, which was absent in our earlier functional gene-based analysis, and revealed a consistent homogenization trend under AS. Although this work is based on biotic and fundamental soil chemical attributes, future studies could extend these findings by directly assessing spatial heterogeneity in soil processes, such as respiration and nutrient processing, as well as in measures of system resilience in mesocosm experiments.

## Supplementary Material

Figure_S1_wraf264

Figure_S2_wraf264

Figure_S3_wraf264

Figure_S4_wraf264

Figure_S5_wraf264

Figure_S6_wraf264

Figure_S7_wraf264

Figure_S8_wraf264

Figure_S9_wraf264

Figure_S10_wraf264

Figure_S11_wraf264

Figure_S12_wraf264

Figure_S13_wraf264

Figure_S14_wraf264

Table_S1_wraf264

Table_S2_wraf264

Table_S3_wraf264

Table_S4_wraf264

Table_S5_wraf264

Table_S6_wraf264

Table_S7_wraf264

Table_S8_wraf264

Supplemental_information_wraf264

## Data Availability

The raw FASTQ data of shotgun sequencing has been deposited into Genome Sequence Archive (GSA) with the accession number of CRA004163, and the raw FASTQ data of amplicon sequencing has been deposited into National Center for Biotechnology Information (NCBI) with the accession number of PRJNA1190147. The pipeline for processing and analyzing the data was implemented in the available GitHub repository (https://github.com/mak3outhill/Land_conversion).
