# Supplementary material for: Land conversion to cropland homogenizes variation in soil biota, gene assemblages, and ecological strategies on local and regional scales
Source: ISME J. 2025 Dec 1;19(1):wraf264. doi: 10.1093/ismejo/wraf264 (PMC12746289; doi:10.1093/ismejo/wraf264)
Supplement: Figure_S1_wraf264 [file figure_s1_wraf264.pdf]

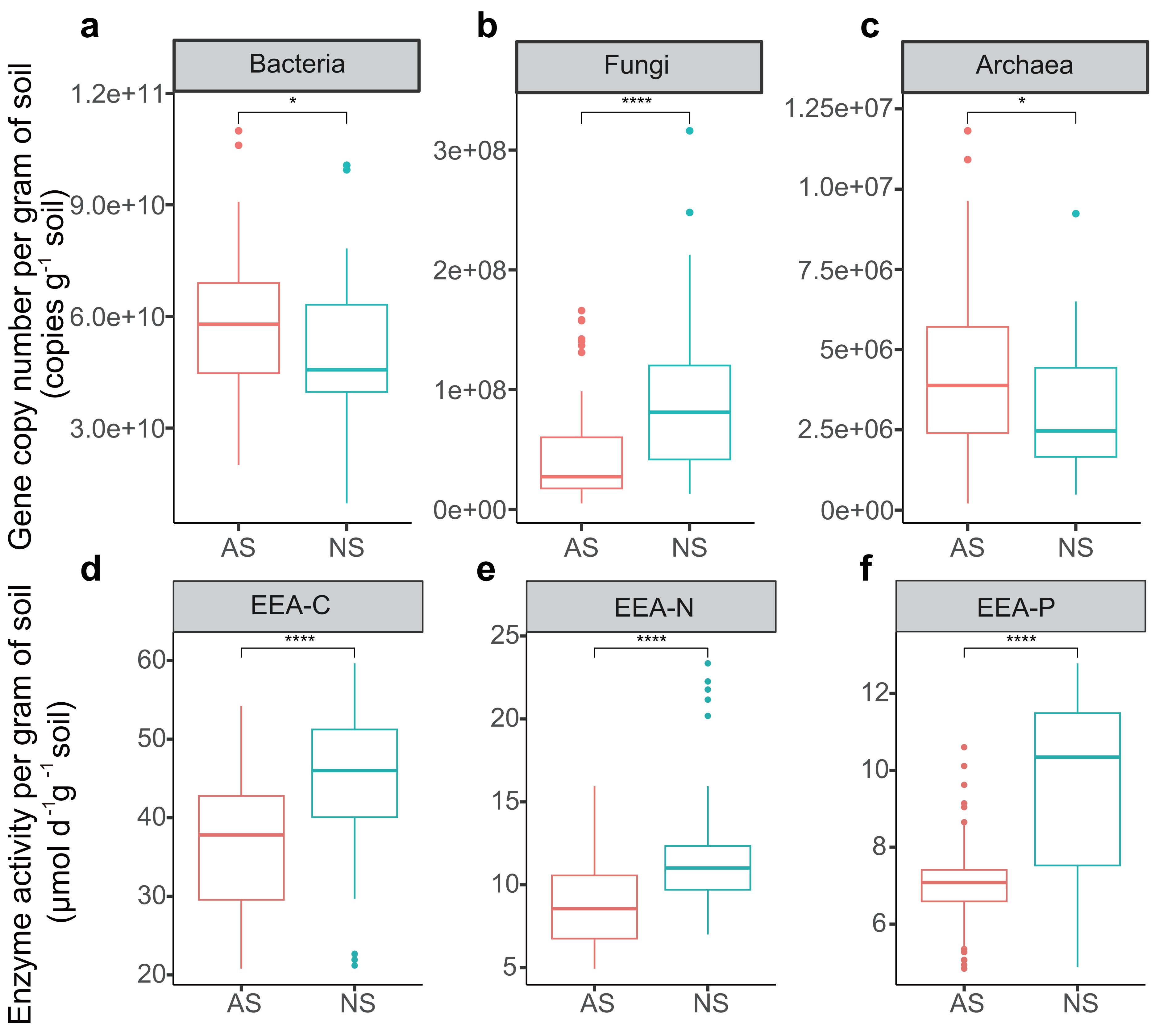
a
Bacteria
b
Fungi
c
Archaea
Gene copy number per gram of soil
(copies g-1 soil)
1.2e+11
9.0e+10
6.0e+10
3.0e+10
3e+08
2e+08
1e+08
0e+00
1.25e+07
1.0e+07
7.5e+06
5e+06
2.5e+06
0e+00
*
****
*
AS
NS
d
EEA-C
e
EEA-N
f
EEA-P
Enzyme activity per gram of soil
(μmol d-1g-1soil)
60
50
40
30
20
25
20
15
10
5
12
10
8
6
****
****
****
AS
NS
