# Supplementary material for: Land conversion to cropland homogenizes variation in soil biota, gene assemblages, and ecological strategies on local and regional scales
Source: ISME J. 2025 Dec 1;19(1):wraf264. doi: 10.1093/ismejo/wraf264 (PMC12746289; doi:10.1093/ismejo/wraf264)
Supplement: Figure_S2_wraf264 [file figure_s2_wraf264.pdf]

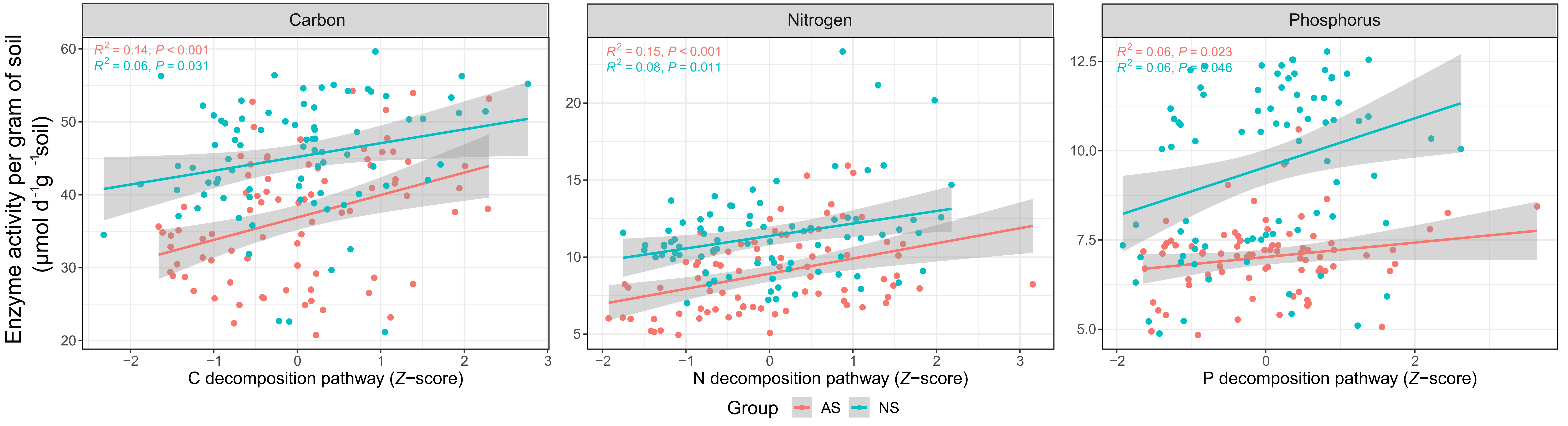

Carbon
Nitrogen
Phosphorus
R^2 = 0.14, P < 0.001
R^2 = 0.06, P = 0.031
R^2 = 0.15, P < 0.001
R^2 = 0.08, P = 0.011
R^2 = 0.06, P = 0.023
R^2 = 0.06, P = 0.046
Enzyme activity per gram of soil
(μmol $d^{-1}g^{-1}$soil)
C decomposition pathway (Z−score)
N decomposition pathway (Z−score)
P decomposition pathway (Z−score)
Group
AS
NS
