# Supplementary figures and images for: Land conversion to cropland homogenizes variation in soil biota, gene assemblages, and ecological strategies on local and regional scales
Source: ISME J. 2025 Dec 1;19(1):wraf264. doi: 10.1093/ismejo/wraf264 (PMC12746289; doi:10.1093/ismejo/wraf264)

**a**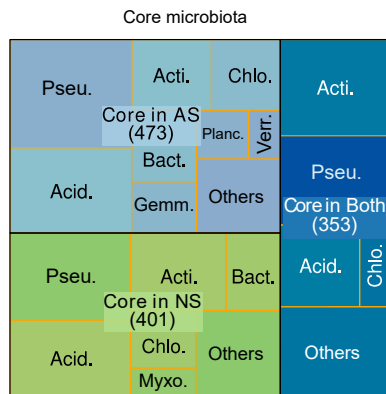**b**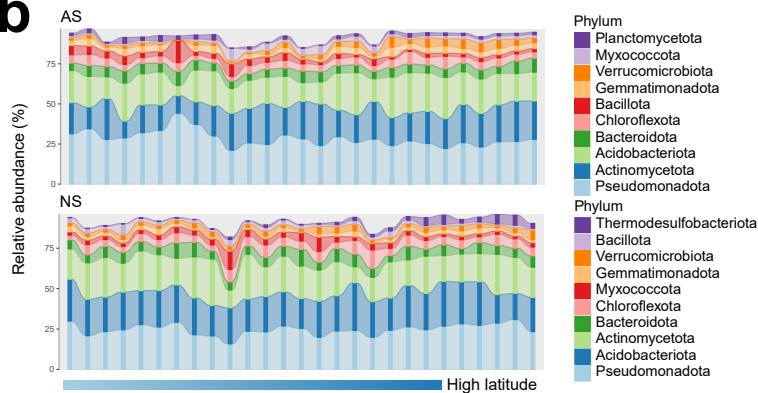**c**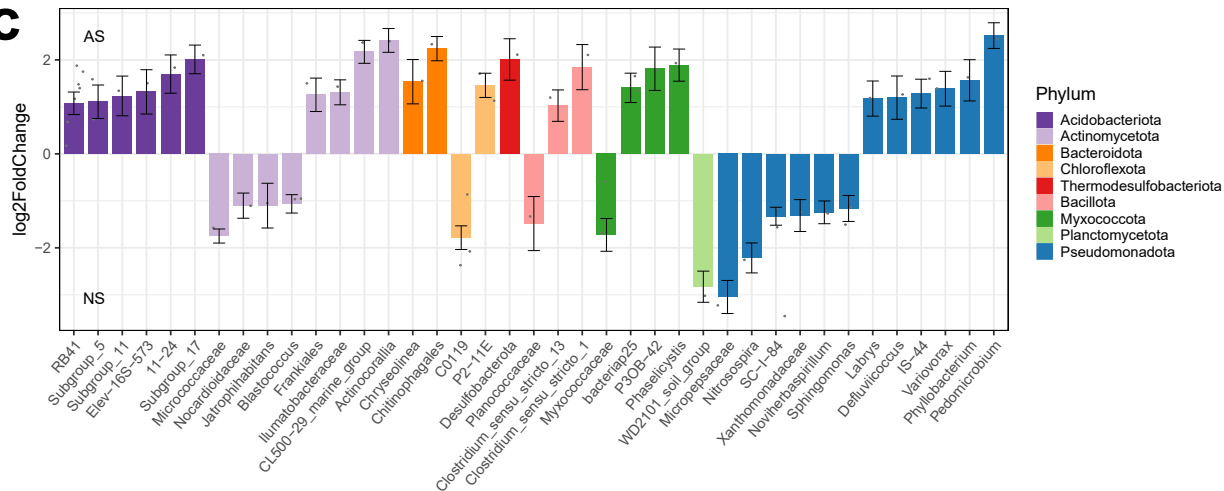

Supplement: Figure_S3_wraf264 [file figure_s3_wraf264.pdf]

**a**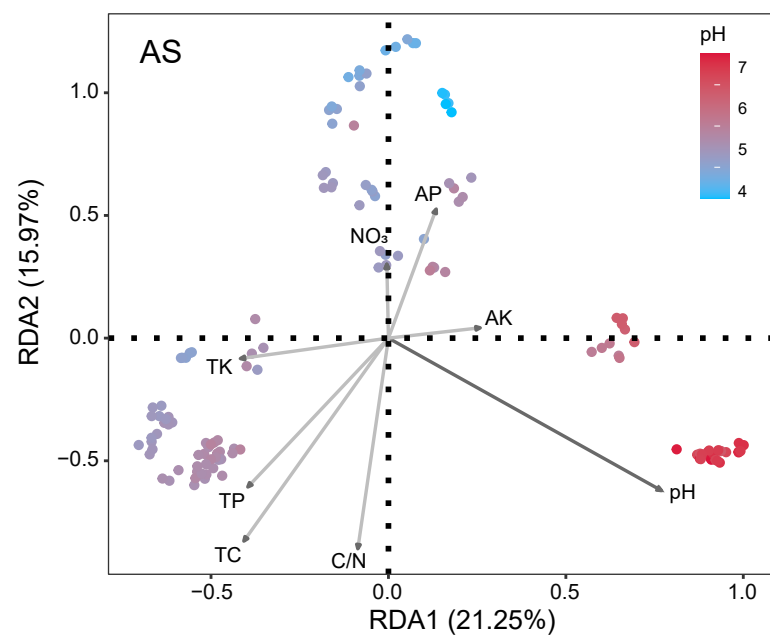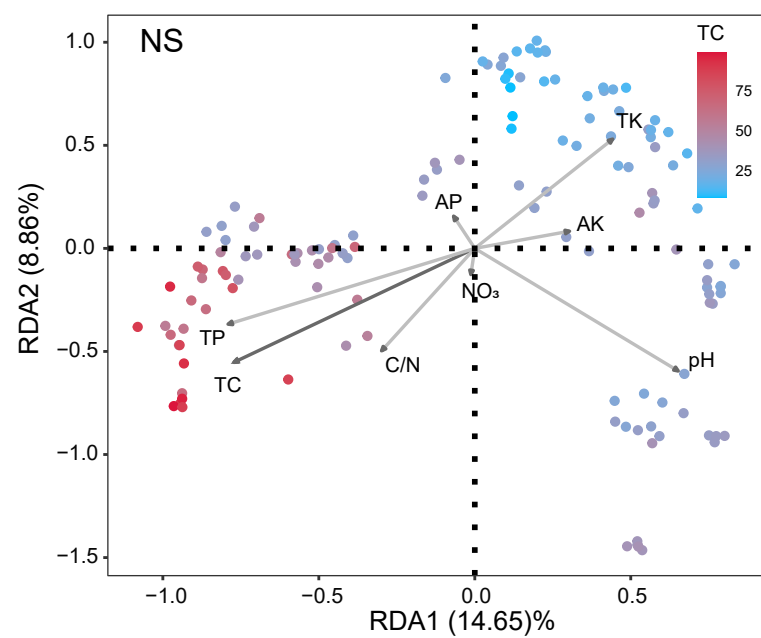**b**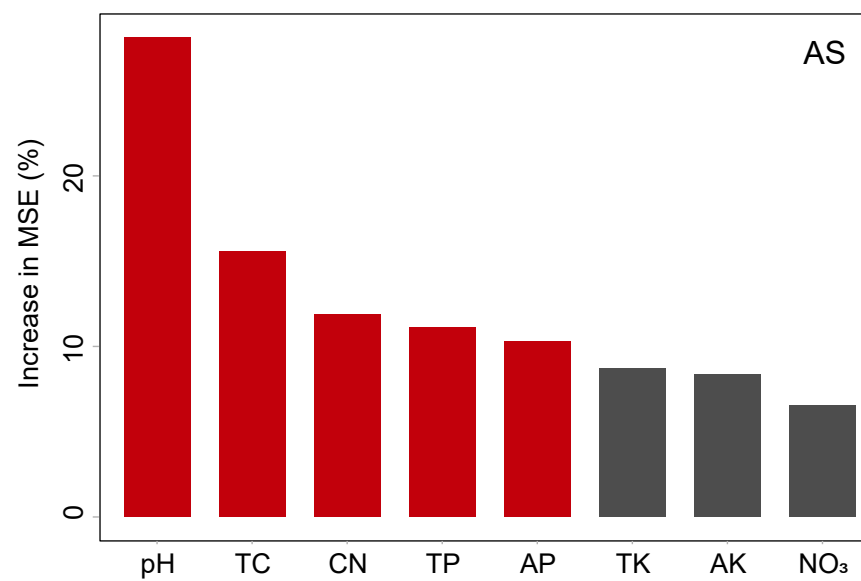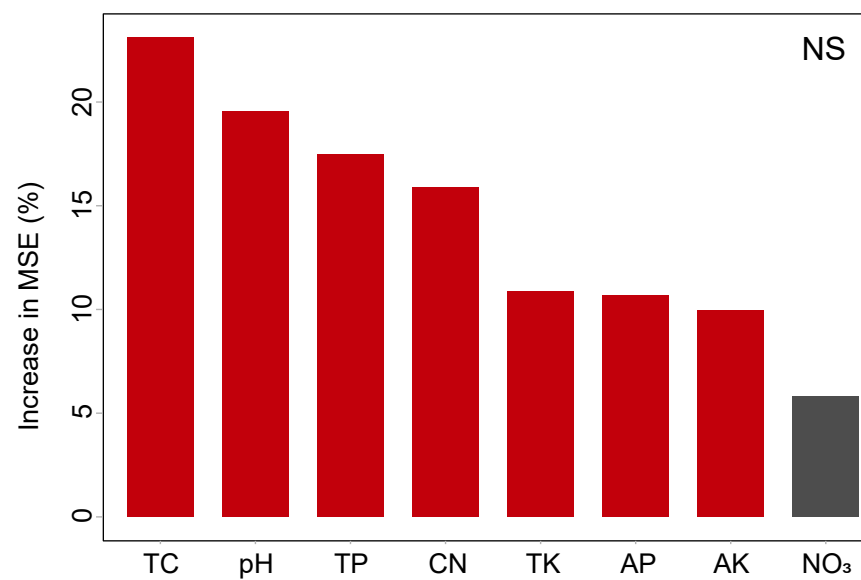**c**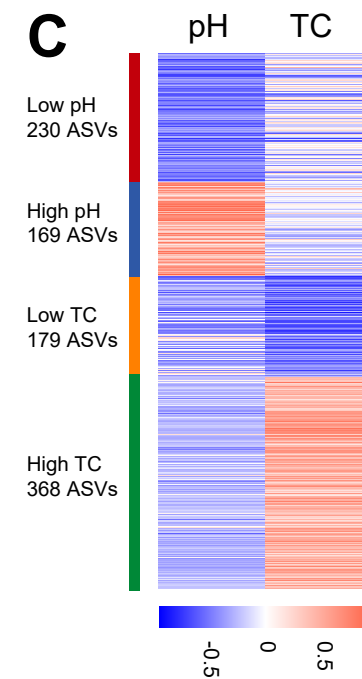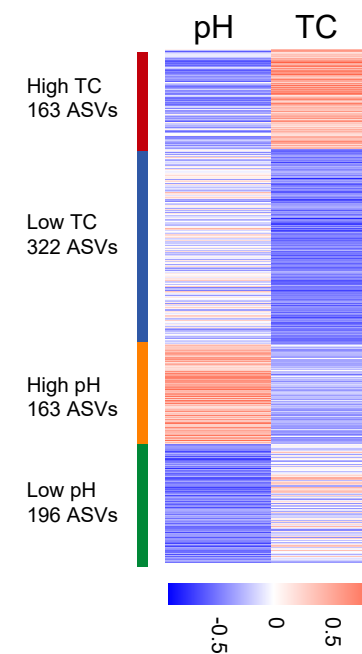

Supplement: Figure_S4_wraf264 [file figure_s4_wraf264.pdf]

**a**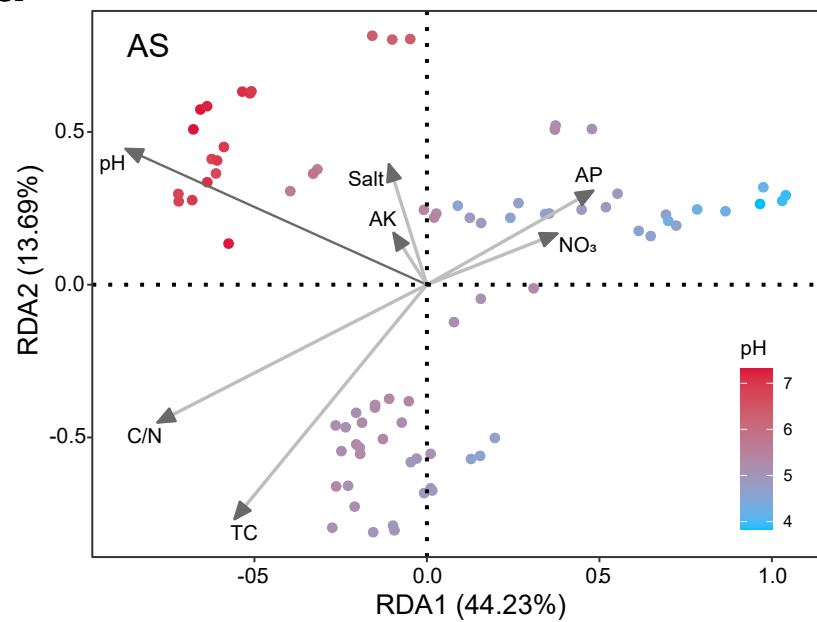**b**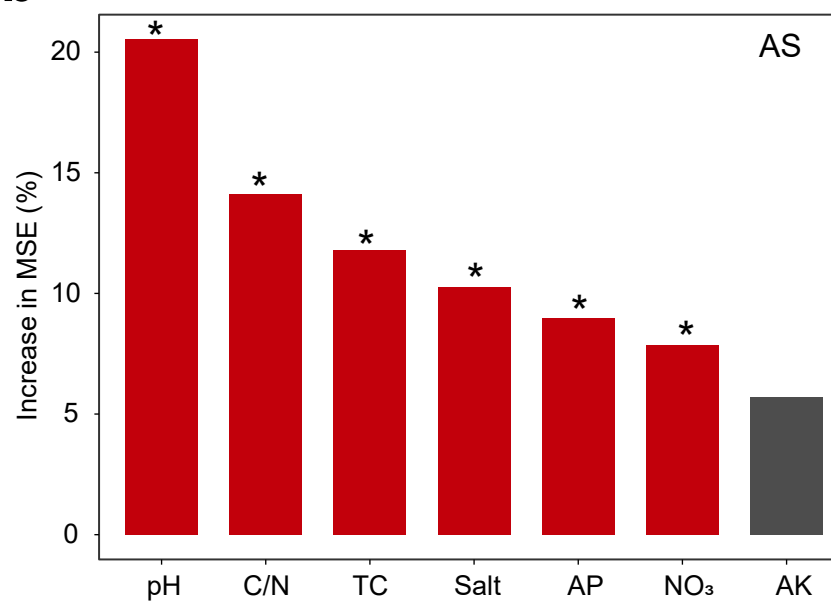**c**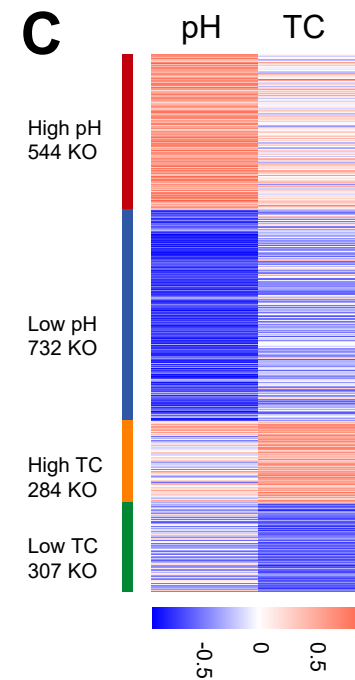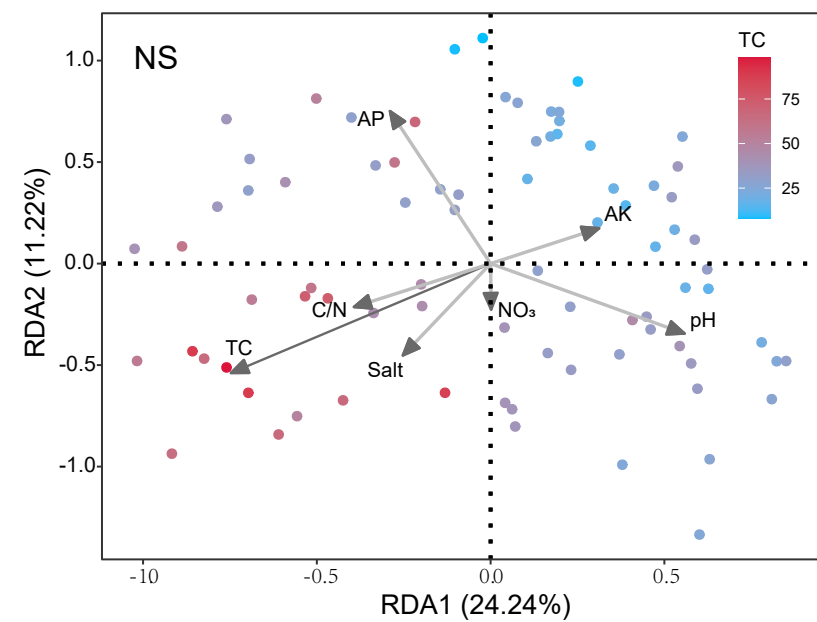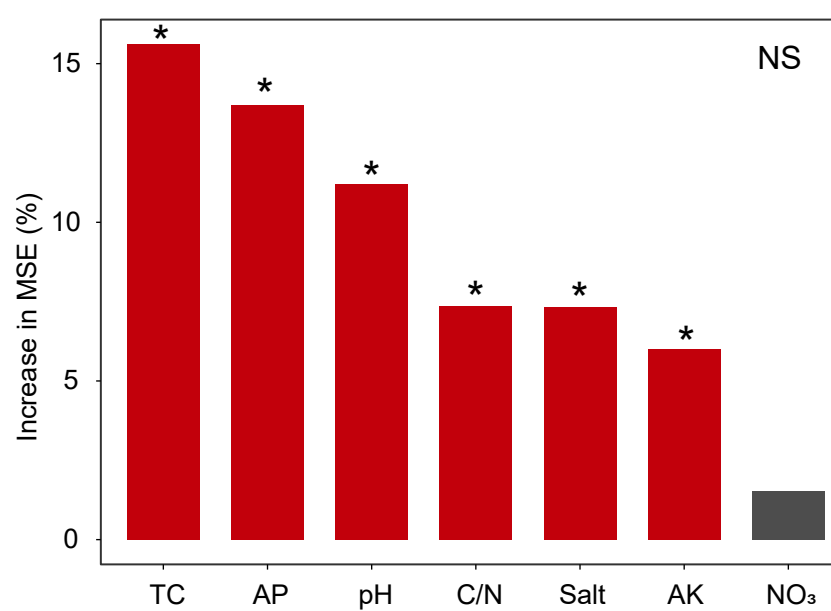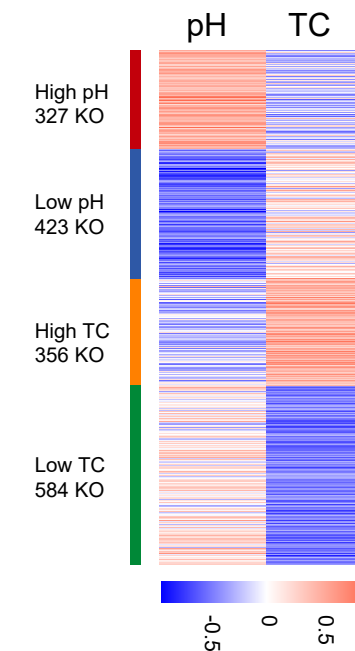

Supplement: Figure_S5_wraf264 [file figure_s5_wraf264.pdf]

Distance to centroid (Bray–Curtis)  
16S rRNA

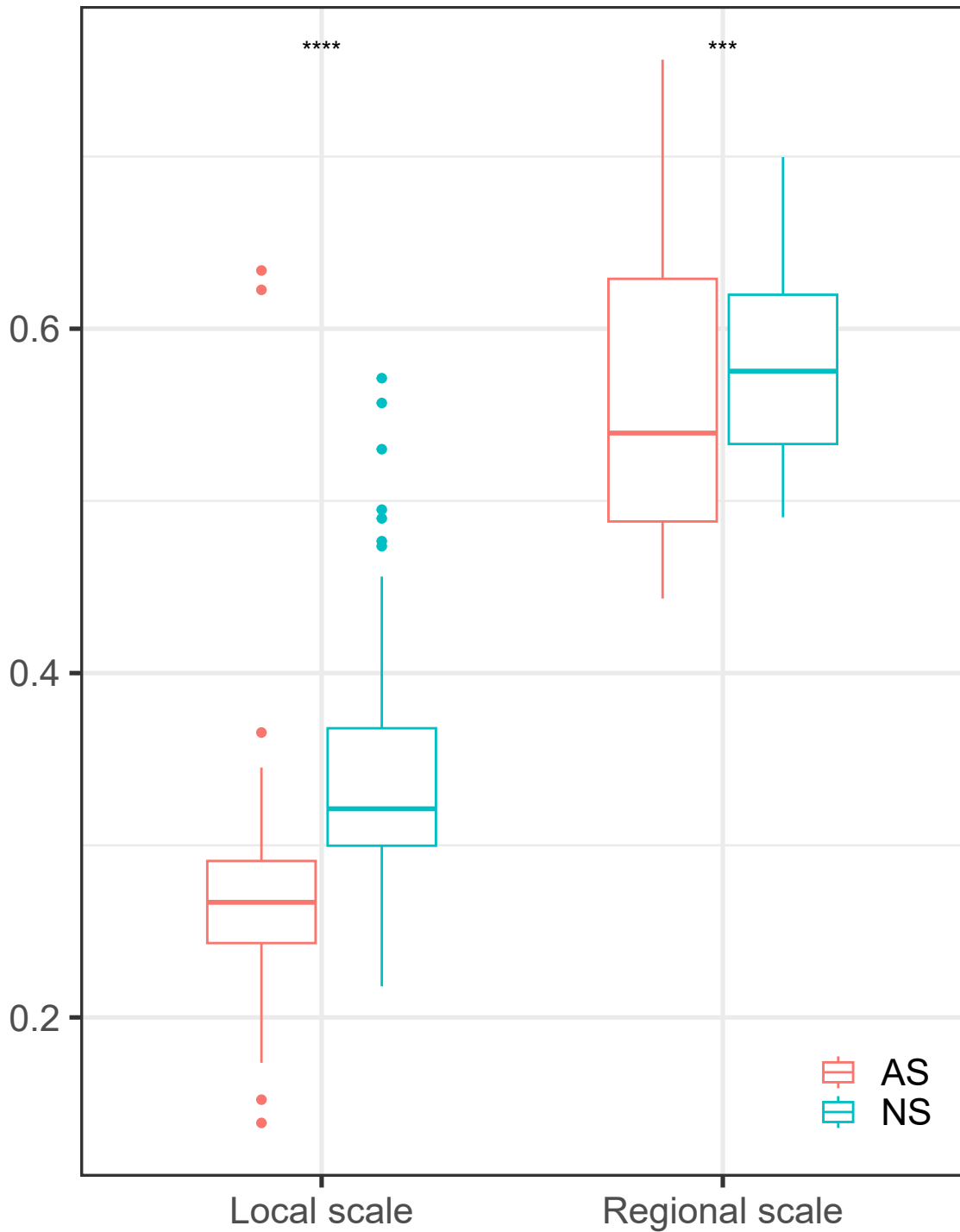

Supplement: Figure_S7_wraf264 [file figure_s7_wraf264.pdf]

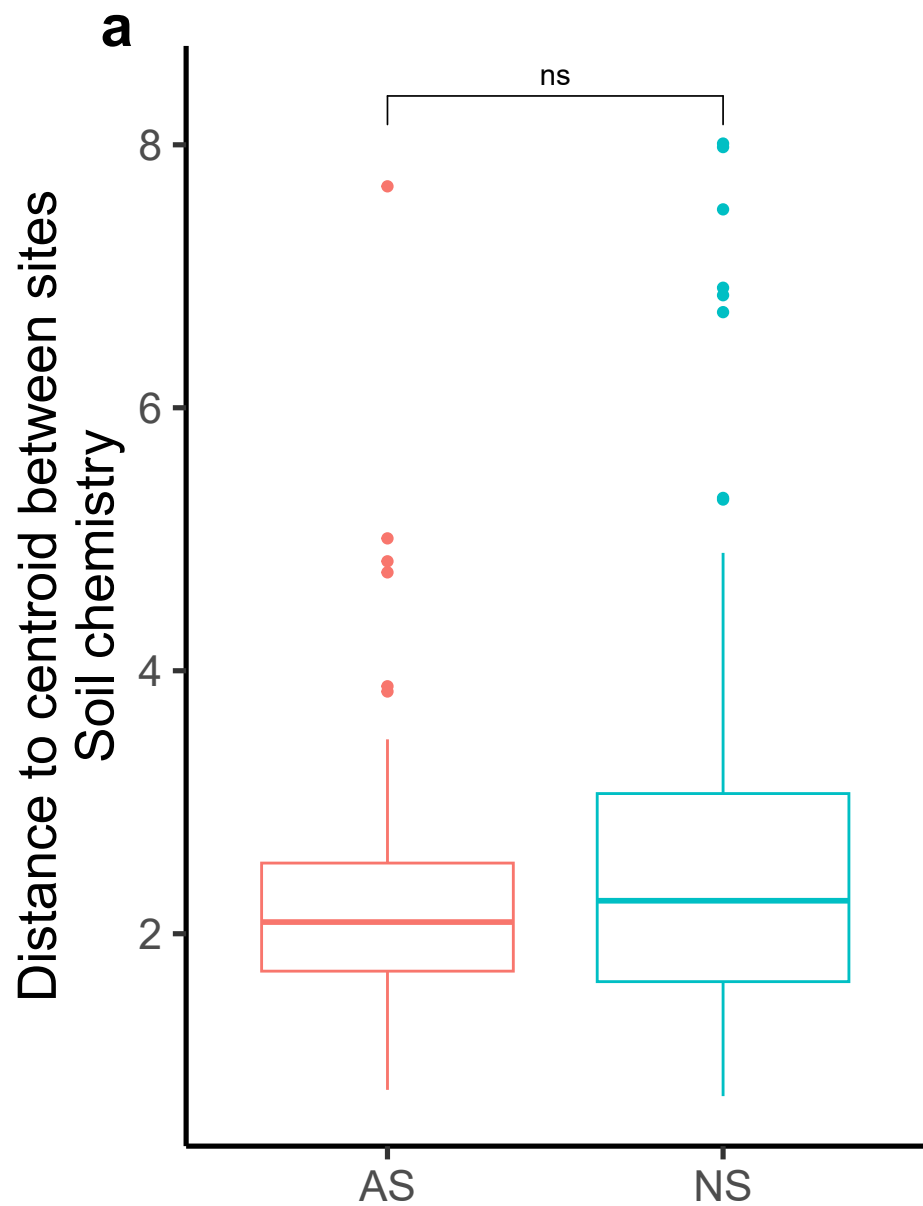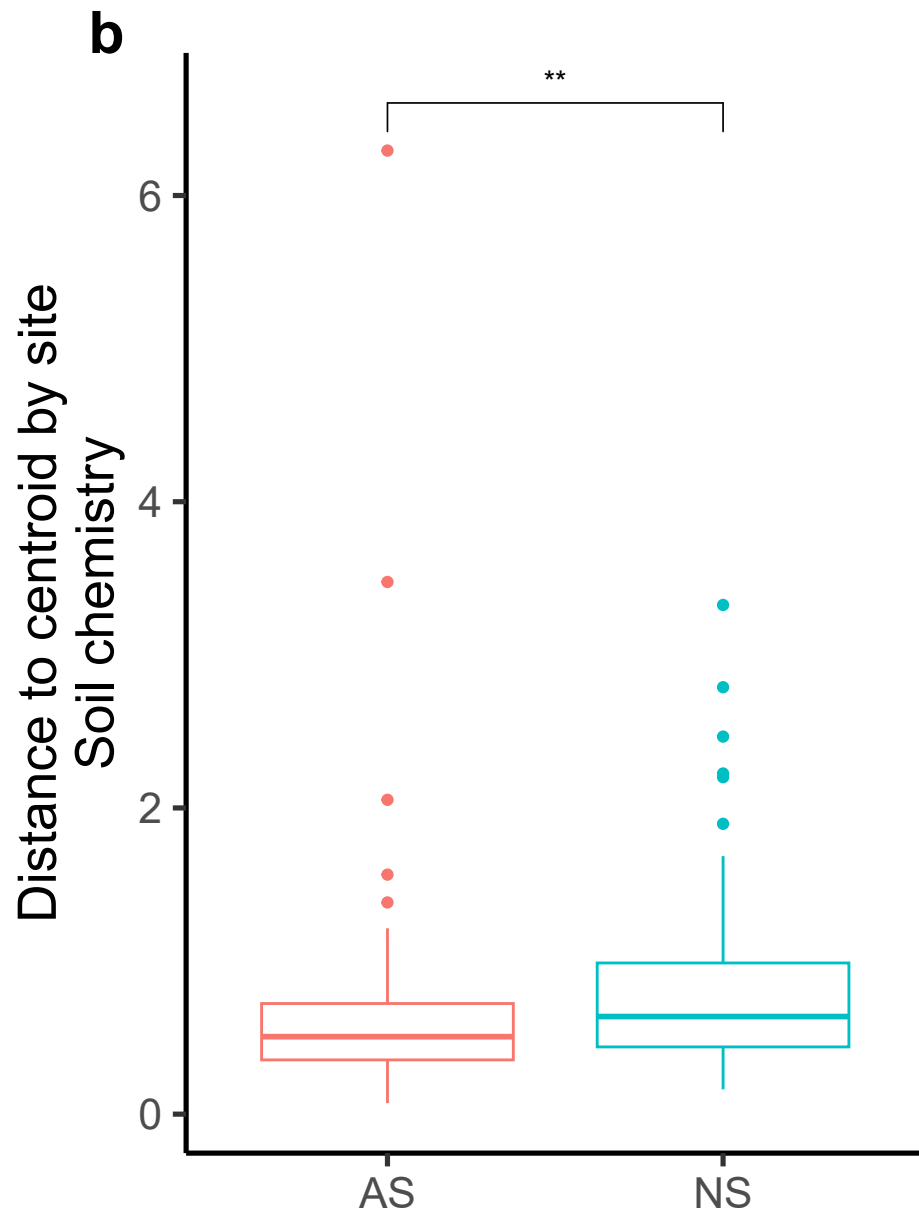

Supplement: Figure_S8_wraf264 [file figure_s8_wraf264.pdf]

Distance to centroid between sites  
Soil chemistry

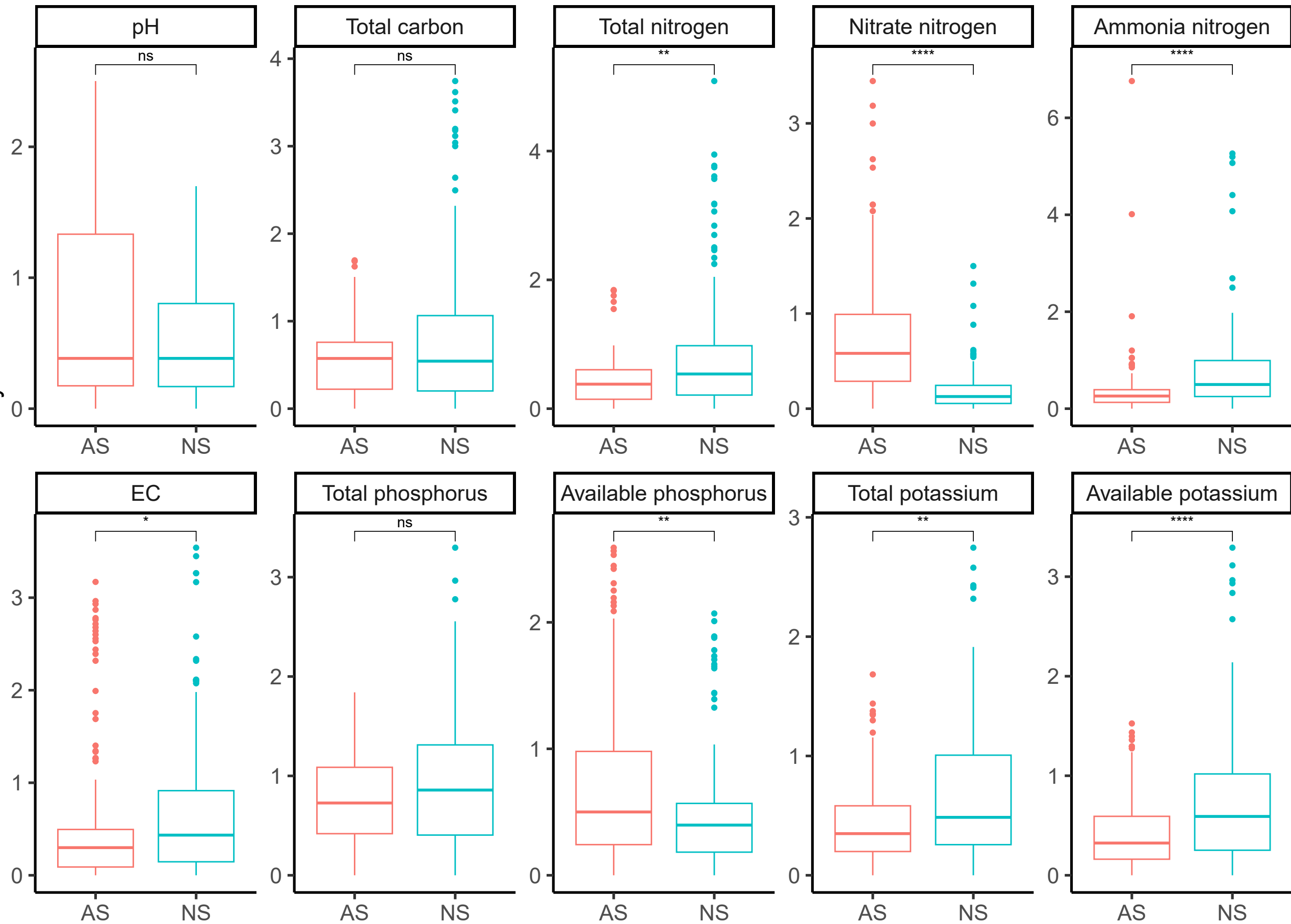

Supplement: Figure_S9_wraf264 [file figure_s9_wraf264.pdf]

Distance to centroid by site  
Soil chemistry

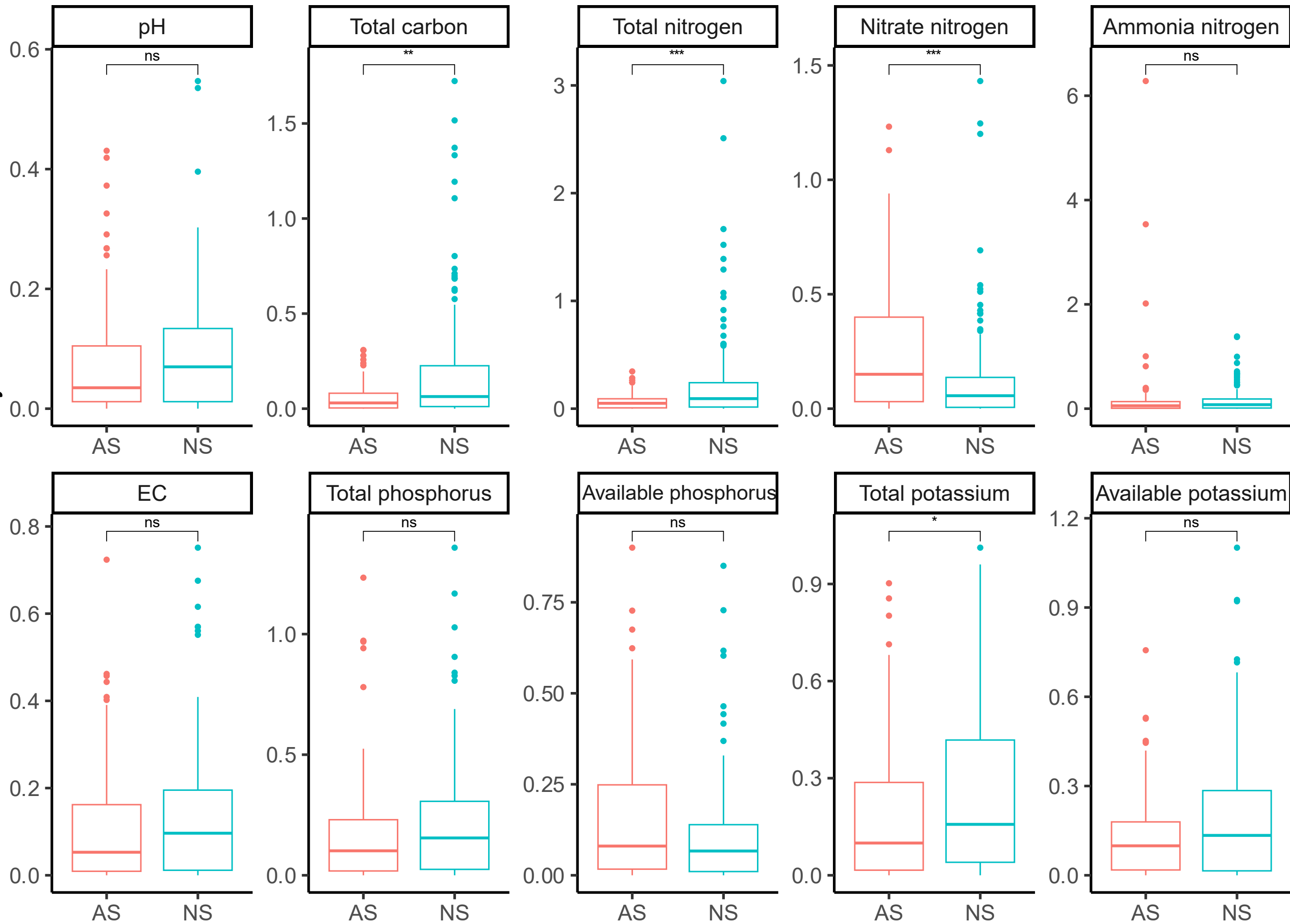

Supplement: Figure_S10_wraf264 [file figure_s10_wraf264.pdf]

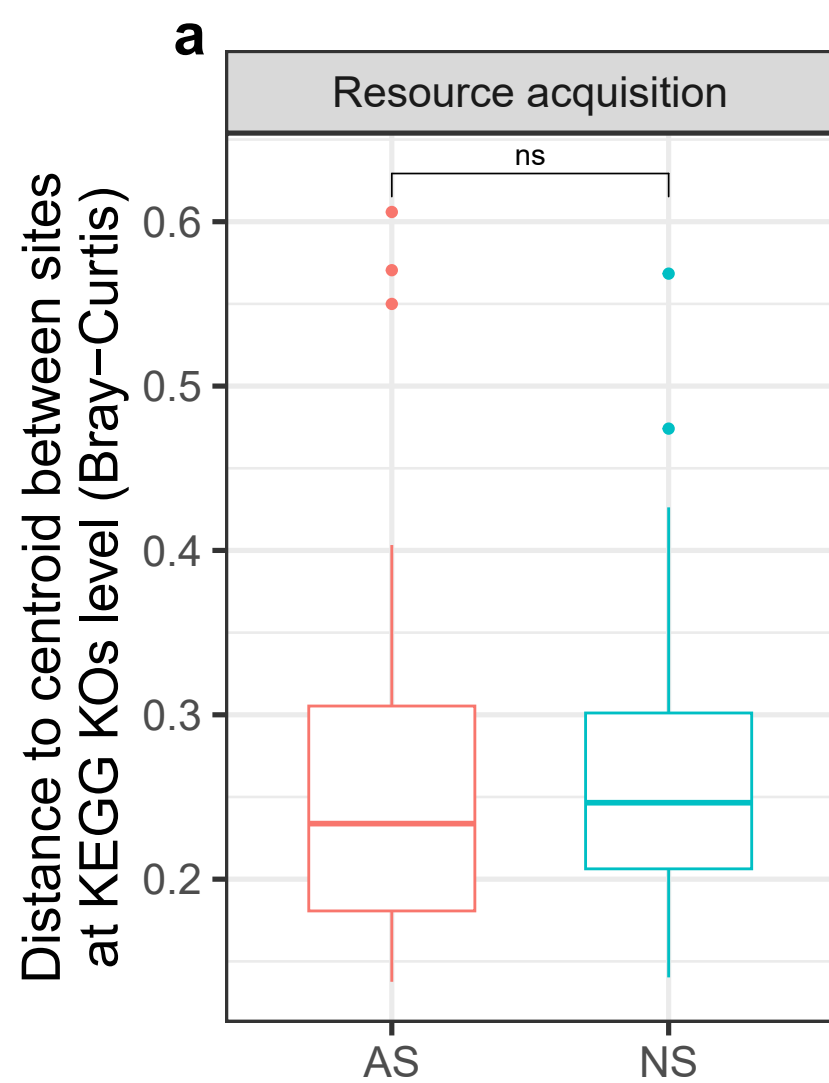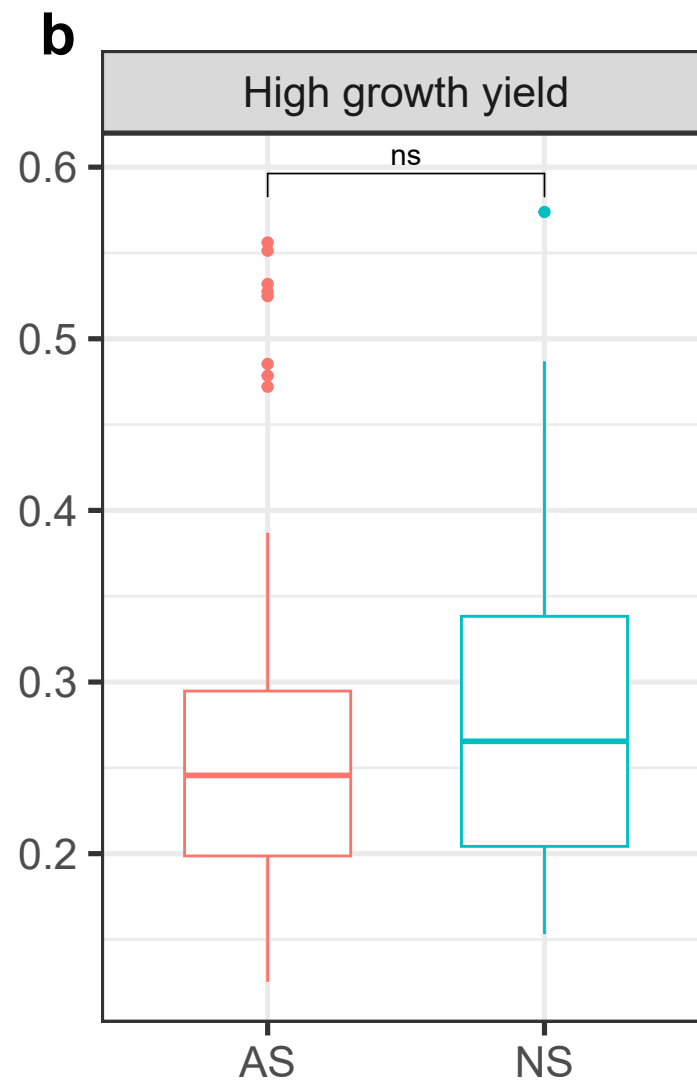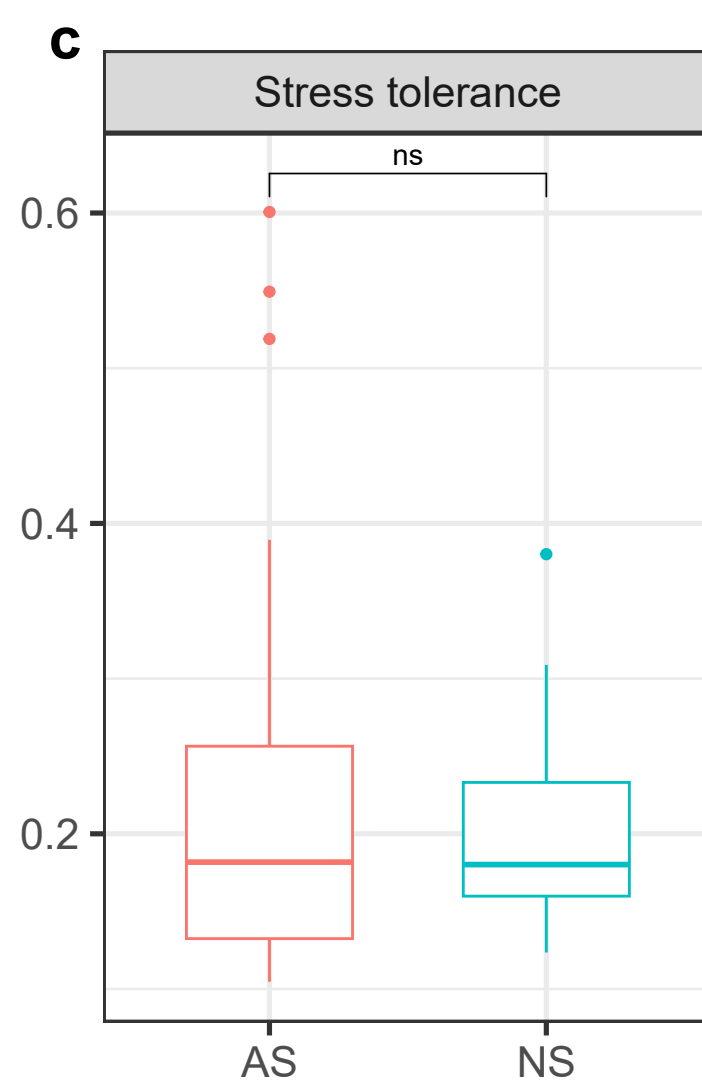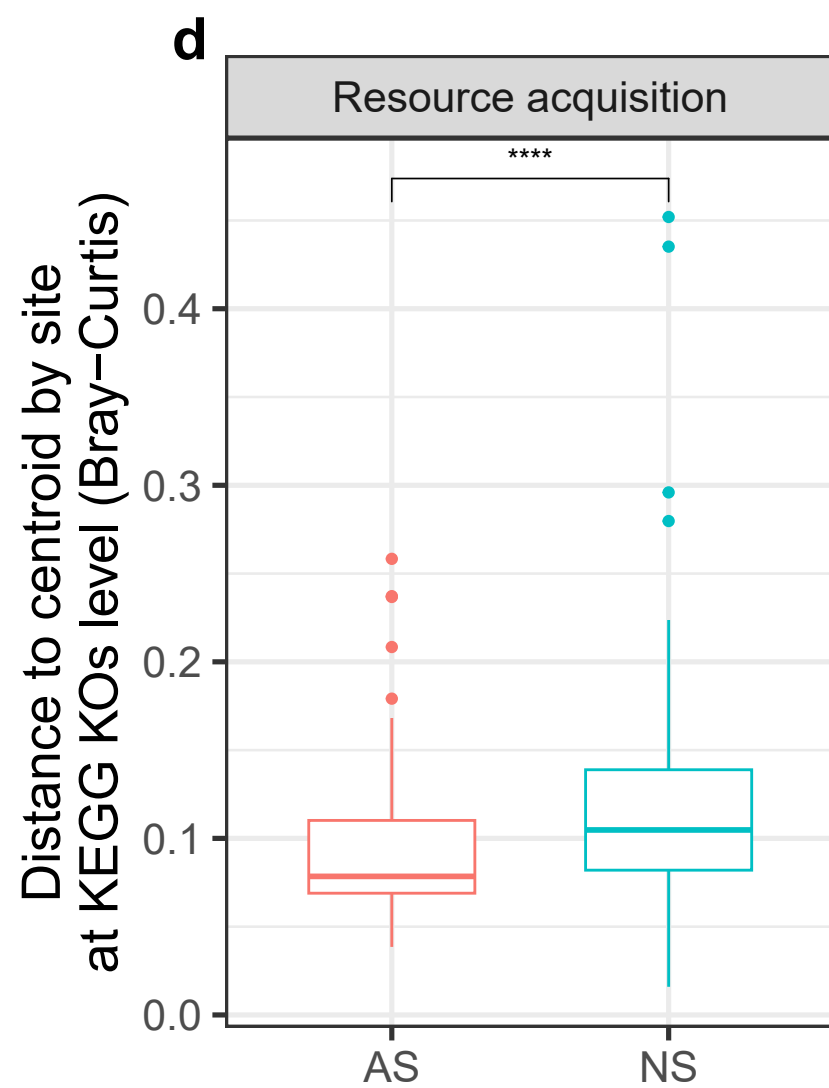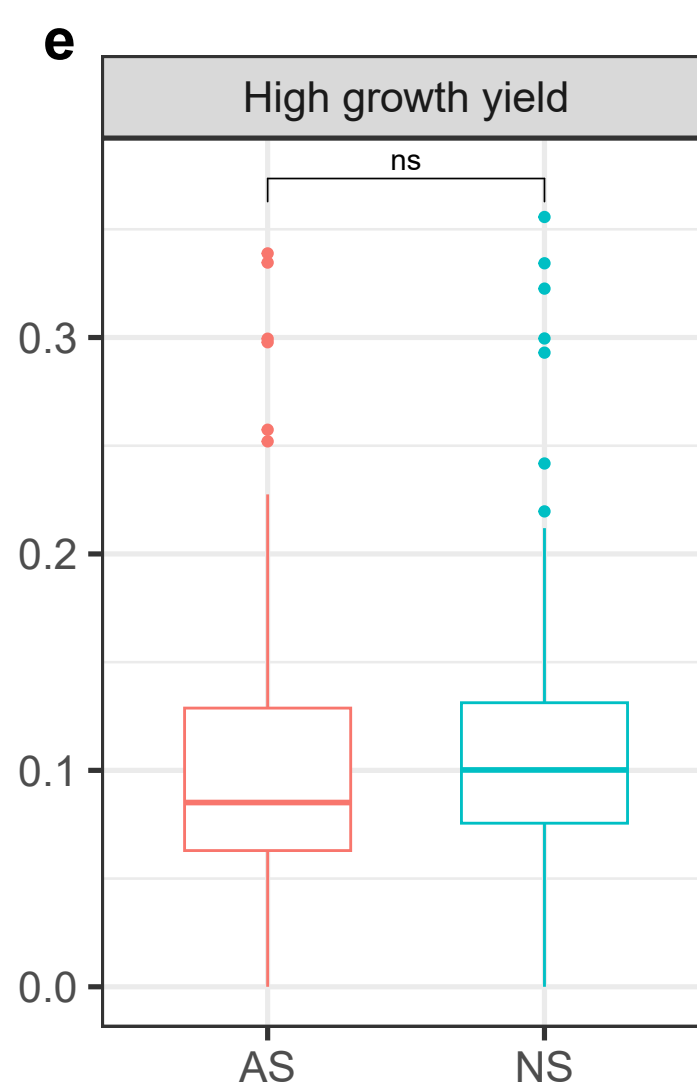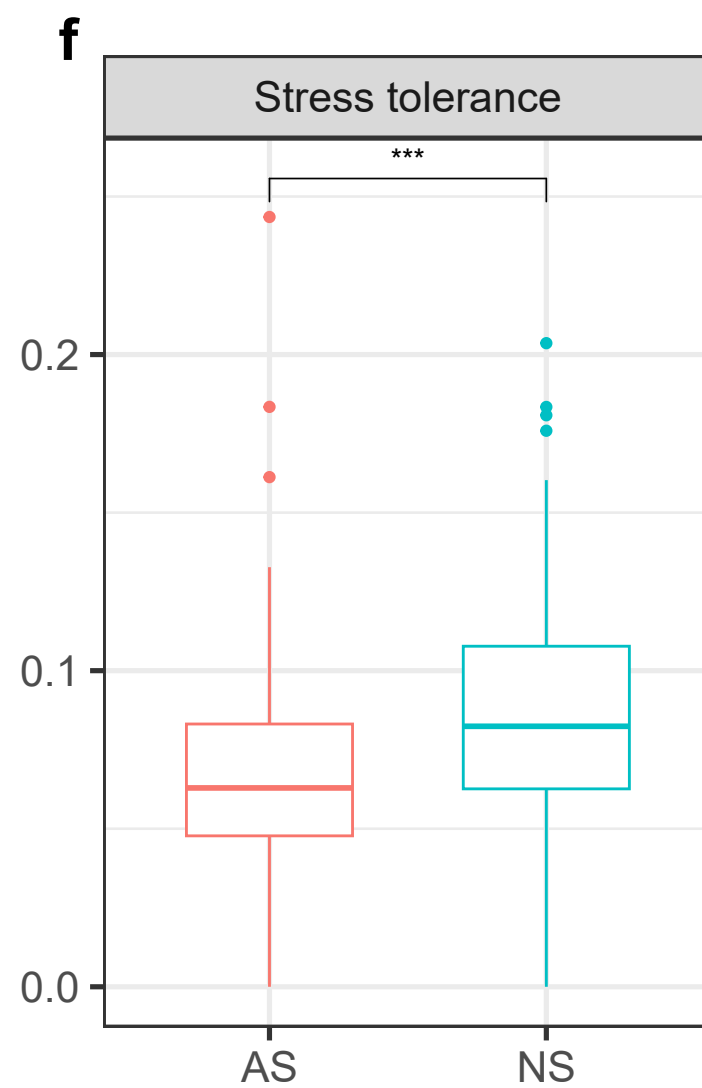

Supplement: Figure_S11_wraf264 [file figure_s11_wraf264.pdf]

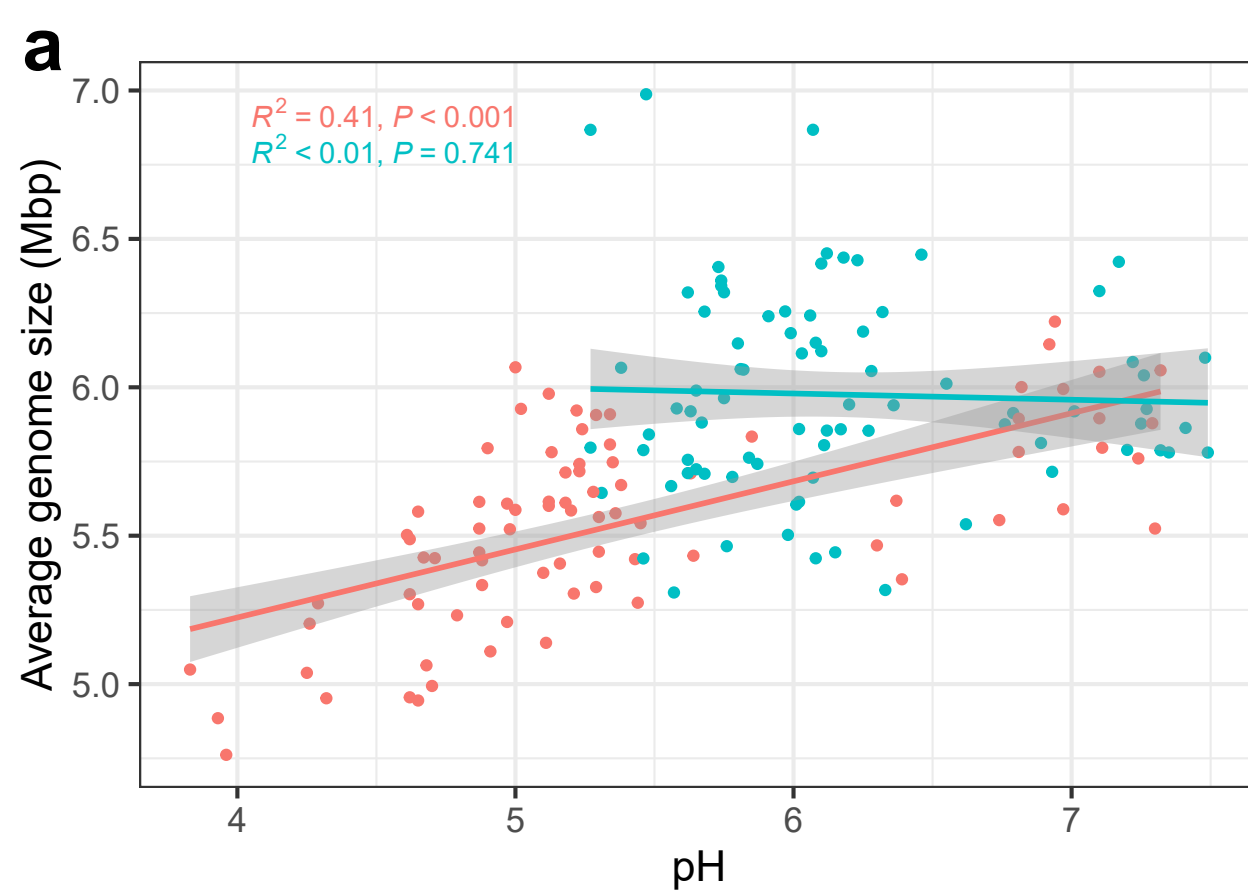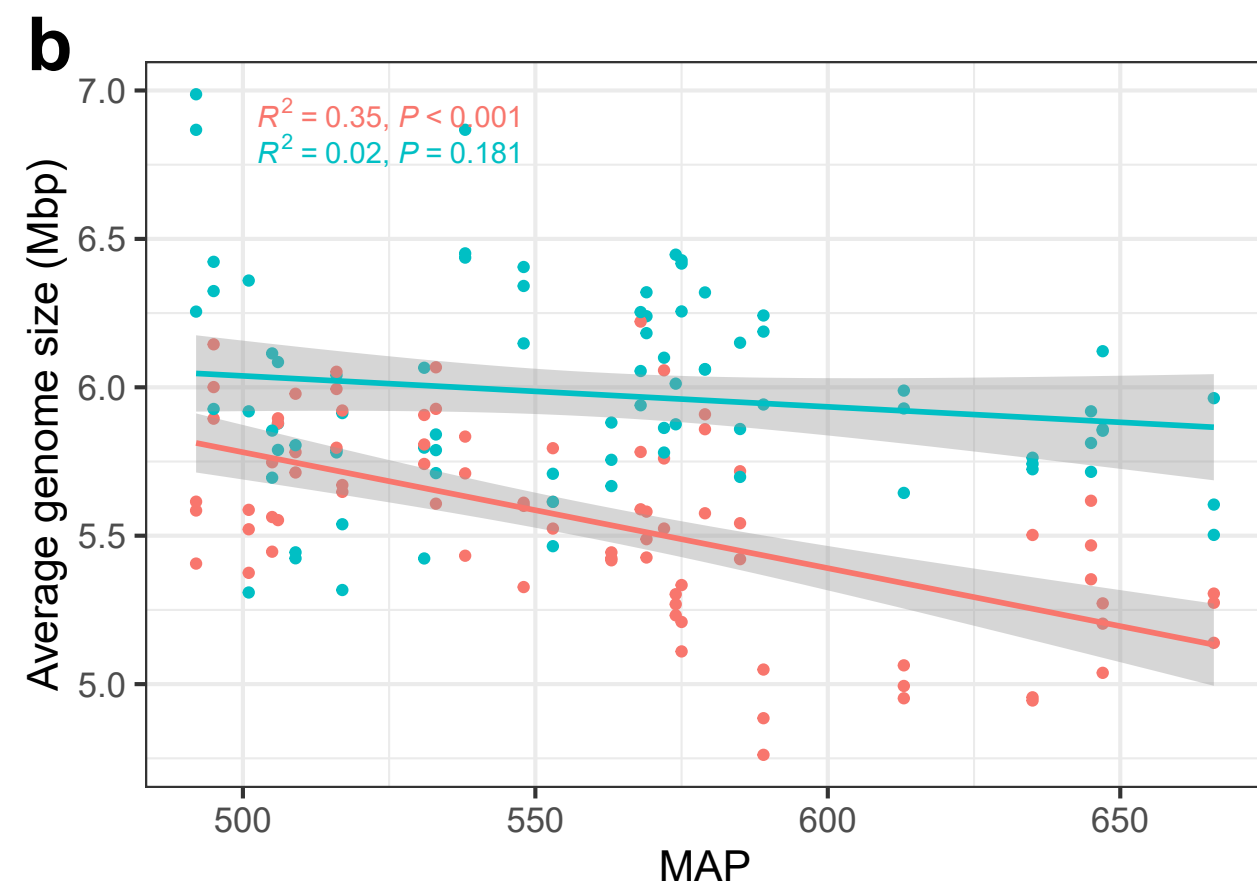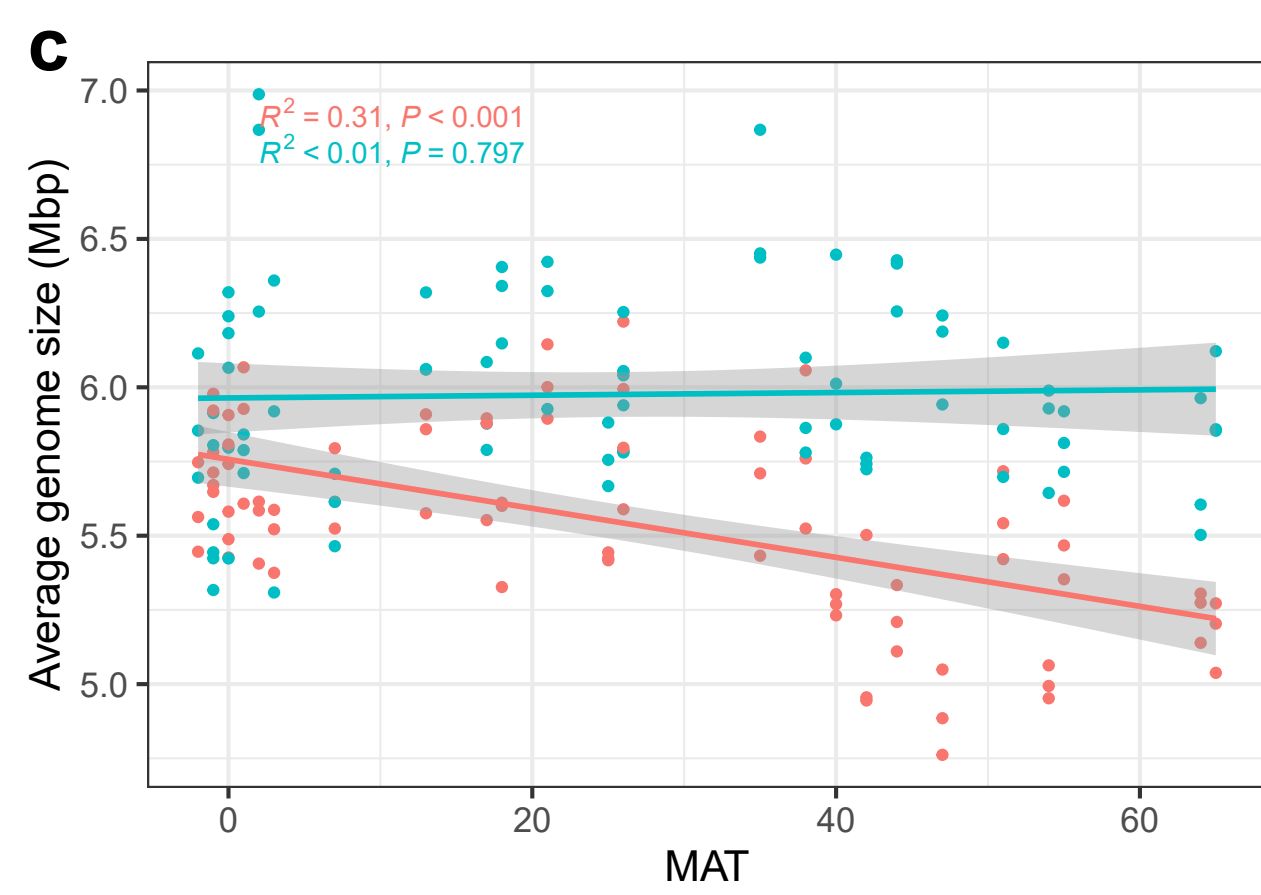

Group AS NS

Supplement: Figure_S12_wraf264 [file figure_s12_wraf264.pdf]

Average genome size (Mbp)

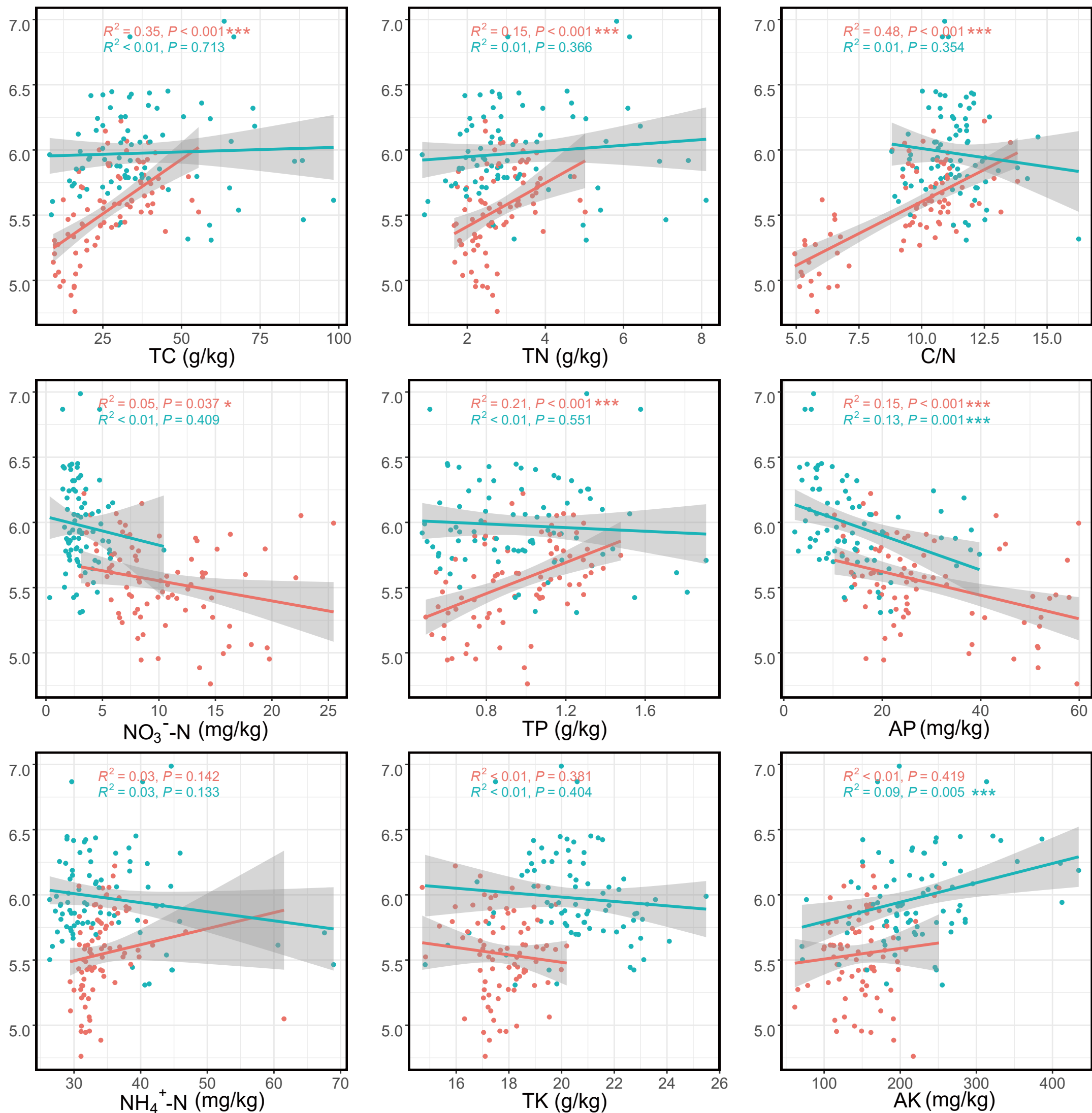

Group AS NS

Supplement: Figure_S13_wraf264 [file figure_s13_wraf264.pdf]

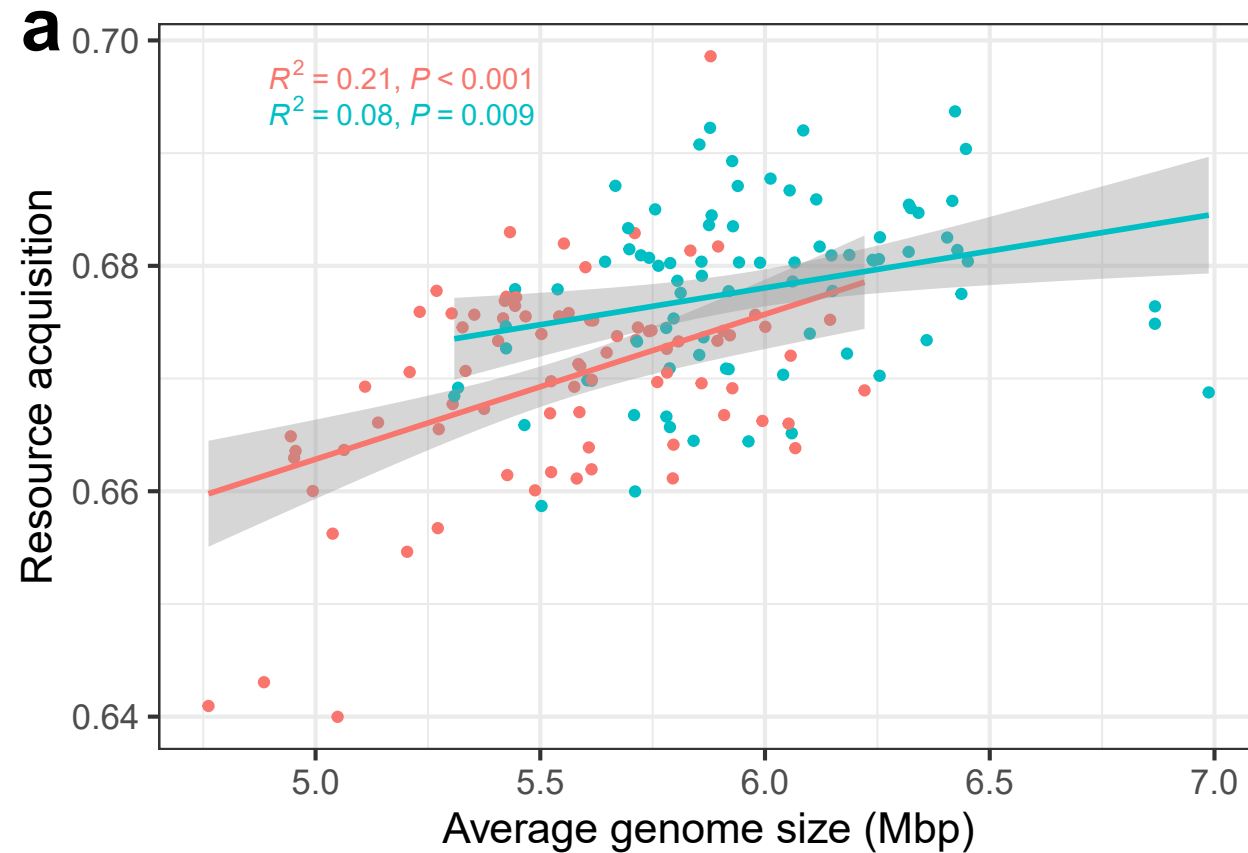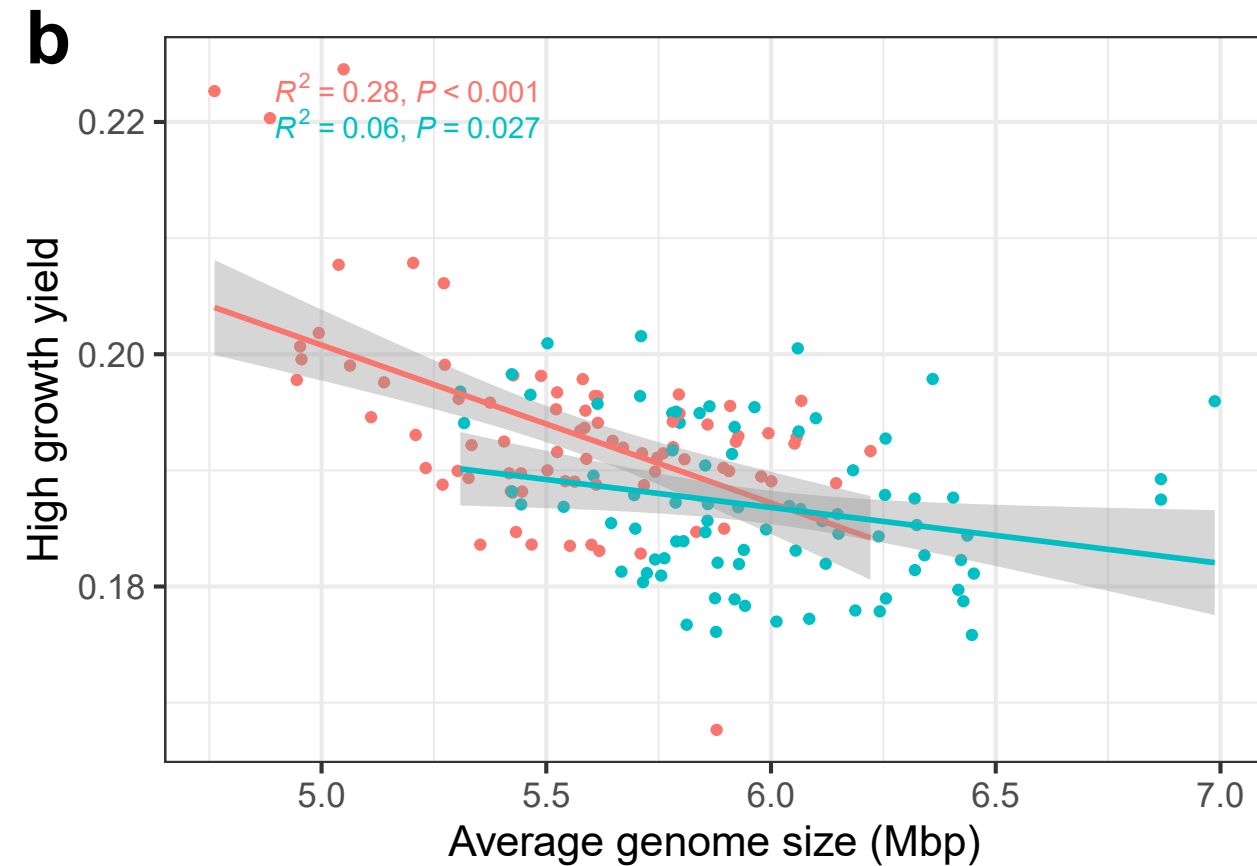

Group AS NS

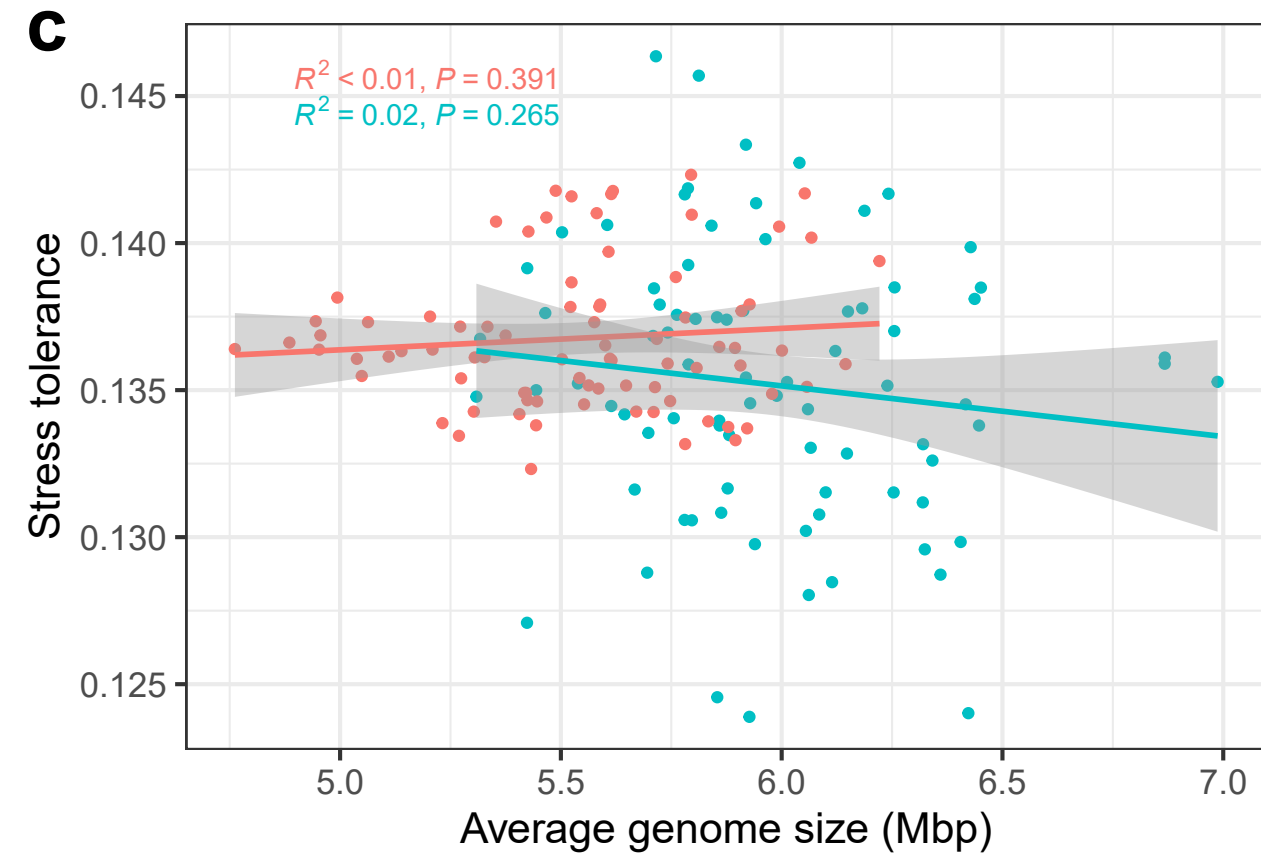

Supplement: Figure_S14_wraf264 [file figure_s14_wraf264.pdf]
