# Supplementary material for: Land conversion to cropland homogenizes variation in soil biota, gene assemblages, and ecological strategies on local and regional scales
Source: ISME J. 2025 Dec 1;19(1):wraf264. doi: 10.1093/ismejo/wraf264 (PMC12746289; doi:10.1093/ismejo/wraf264)
Supplement: Figure_S6_wraf264 [file figure_s6_wraf264.pdf]

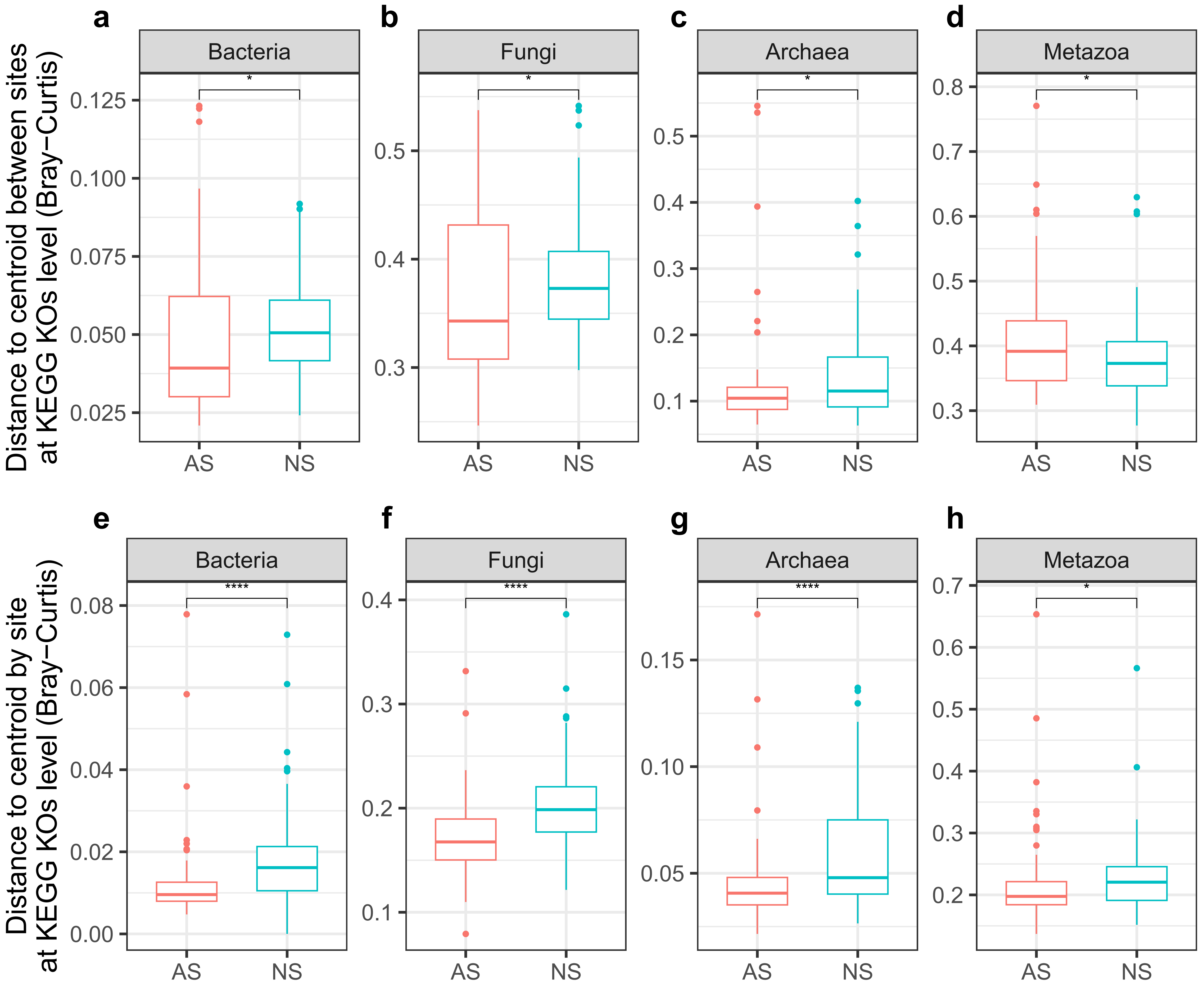
a
b
c
d
Bacteria
Fungi
Archaea
Metazoa
Distance to centroid between sites at KEGG KOs level (Bray−Curtis)
0.125
0.100
0.075
0.050
0.025
0.5
0.4
0.3
0.5
0.4
0.3
0.2
0.1
0.8
0.7
0.6
0.5
0.4
0.3
*
*
*
*
AS
NS
e
f
g
h
Bacteria
Fungi
Archaea
Metazoa
Distance to centroid by site at KEGG KOs level (Bray−Curtis)
0.08
0.06
0.04
0.02
0.00
0.4
0.3
0.2
0.1
0.15
0.10
0.05
0.7
0.6
0.5
0.4
0.3
0.2
****
****
****
*
AS
NS
