# Supplementary material for: Land conversion to cropland homogenizes variation in soil biota, gene assemblages, and ecological strategies on local and regional scales
Source: ISME J. 2025 Dec 1;19(1):wraf264. doi: 10.1093/ismejo/wraf264 (PMC12746289; doi:10.1093/ismejo/wraf264)
Supplement: Supplemental_information_wraf264 [file supplemental_information_wraf264.docx]

**Supplemental Figure and Table legends:**

**Figure S1.** The abundances of bacterial (a), fungal (b), and archaeal (c) communities in agricultural (AS) and natural soils (NS). The extracellular enzyme activity associated with the degradation of carbon (d), nitrogen (e), and phosphorus (f) in AS and NS.

**Fig S2.** The correlation analysis between the abundances of genes involved in carbon-, nitrogen-, and phosphorus-degradation pathways and the activities of their respective enzymes.

**Fig S3.** The composition of core ASVs among agricultural soils (AS) and natural soils (NS). (a) Shared and unique core ASVs between AS and NS. The size of each block represents the core ASVs number. (b) Relative abundances of bacterial phyla across different sampling sites. (c) Differences in relative abundance of bacterial genus between AS and NS. The bar indicates the log_2_fold changes (LFCs) for bacterial genera. Only genera with significant LFCs (*P* < 0.05) were displayed. Colors represent the different phyla, and dots represent the LFCs of individual ASVs.

**Fig S4.** Key environmental drivers and ecological clusters of bacterial amplicon sequence variants (ASVs) in AS and NS. (a) Redundancy analysis (RDA) showing the primary soil factors regulating the bacterial communities in AS and NS, respectively. Sample points are colored based on soil pH gradients in AS and total carbon (TC) gradients in NS, with the color scale ranging from high (red) to low (blue) values. (b) Predictive importance of environmental factors on bacterial ASV beta-diversity, as quantified by random forest analysis using the percentage increase in mean square error (% IncMSE). (c) Spearman correlation analysis between the relative abundance of each core bacterial taxon and the key environmental drivers (soil pH and TC). Significance levels: **P* < 0.05 and ***P* < 0.01. TC, total carbon content; AP, available phosphorus content; C/N, carbon nitrogen ratio; SC, salt content; NO_3_^-^-N, nitrate-nitrogen; AK, available potassium content.

**Fig S5.** Key environmental drivers and ecological clusters of functional genes in AS and NS. (a) Redundancy analysis (RDA) showing the primary soil factors regulating functional gene communities in AS and NS, respectively. Sample points are colored based on soil pH gradients in AS and total carbon (TC) gradients in NS, with the color scale ranging from high (red) to low (blue) values. (b) Predictive importance of environmental factors on functional gene beta-diversity, as quantified by random forest analysis using the percentage increase in mean square error (% IncMSE). (c) Spearman correlation analysis between the relative abundance of each core functional gene and the key environmental drivers (soil pH and TC). Significance levels: **P* < 0.05 and ***P* < 0.01. TC, total carbon content; AP, available phosphorus content; C/N, carbon nitrogen ratio; SC, salt content; NO_3_^-^-N, nitrate-nitrogen; AK, available potassium content.

**Figure S6**. Differences in homogeneity at KEGG KOs level of the soil bacterial, fungal, archaeal and metazoal communities at the inter-site (a-d) and the intra-site (e-h) level. The asterisks indicate the significant difference between those two land use types (**P* < 0.05, *****P* < 0.0001; Wilcox sum rank test).

**Figure S7.** Differences in homogeneity at ASV level of the soil bacterial communities at the inter-site and the intra-site level. The asterisks indicate the significant difference between those two land use types (****P* < 0.001, *****P* < 0.0001; Wilcox sum rank test).

**Figure S8.** Differences in homogeneity of the soil chemistry at the inter-site (a) and the intra-site (b) level. The asterisks indicate the significant difference between those two land use types (***P* < 0.01, ns *P* > 0.05; Wilcox sum rank test).

**Figure S9.** Differences in homogeneity of the individual soil chemistry at the inter-site level. The asterisks indicate the significant difference between those two land use types (**P* < 0.05, **P* < 0.01, *****P* <0.0001, ns *P* > 0.05; Wilcox sum rank test).

**Figure S10.** Differences in homogeneity of the individual soil chemistry at the intra-site level. The asterisks indicate the significant difference between those two land use types (**P* < 0.05, ***P* < 0.01, ****P* < 0.001, ns *P* > 0.05; Wilcox sum rank test).

**Figure S11.** Differences in homogeneity of life history strategies. The asterisks indicate the significant difference between those two land use types (****P* < 0.001, *****P* < 0.0001, ns *P* > 0.05; Wilcox sum rank test).

**Figure S12.** The relationship between the metagenomic average genome size and environmental factors.

**Figure S13.** The relationship between the metagenomic average genome size (AGS) and soil chemical properties in AS and NS.

**Figure S14.** The relationship between the metagenomic average genome size and relative abundance of life history strategies.

**Table S1.** Soil sampling site locations and chemical fertilization for the agricultural soils.

**Table S2.** Soil chemical properties, climate information, and average genome size used for this study.

**Table S3.** The bacterial taxa showed significant correlations with pH and total carbon in AS and NS.

**Table S4.** The functional genes showed significant correlations with pH and total carbon in AS and NS.

**Table S5.** Information for functional richness at each KEGG level used in this study.

**Table S6.** Network properties of bacterial communities.

**Table S7.** Node properties of bacterial network in AS.

**Table S8.** Node properties of bacterial network in NS.
